# Novel antibody reagents for characterization of drug- and tumor microenvironment-induced changes in epithelial-mesenchymal transition and cancer stem cells

**DOI:** 10.1371/journal.pone.0199361

**Published:** 2018-06-21

**Authors:** Tony Navas, Thomas D. Pfister, Simona Colantonio, Amina Aziz, Lynda Dieckman, Richard G. Saul, Jan Kaczmarczyk, Suzanne Borgel, Sergio Y. Alcoser, Melinda G. Hollingshead, Young H. Lee, Donald P. Bottaro, Tara Hiltke, Gordon Whiteley, Naoko Takebe, Robert J. Kinders, Ralph E. Parchment, Joseph E. Tomaszewski, James H. Doroshow

**Affiliations:** 1 Clinical Pharmacodynamics Biomarker Program, Applied/Developmental Research Directorate, Frederick National Laboratory for Cancer Research sponsored by the National Cancer Institute, Frederick, Maryland, United States of America; 2 Antibody Characterization Laboratory, Frederick National Laboratory for Cancer Research sponsored by the National Cancer Institute, Frederick, Maryland, United States of America; 3 Biosciences Division, Argonne National Laboratory, Argonne, Illinois, United States of America; 4 Biological Testing Branch, Developmental Therapeutics Program, National Cancer Institute at Frederick, Frederick, Maryland, United States of America; 5 Urologic Oncology Branch, Center for Cancer Research, National Cancer Institute, Bethesda, Maryland, United States of America; 6 Office of Cancer Clinical Proteomics Research, Center for Strategic Scientific Initiatives, National Cancer Institute, Bethesda, Maryland, United States of America; 7 Division of Cancer Treatment and Diagnosis, National Cancer Institute, Bethesda, Maryland, United States of America; 8 Center for Cancer Research, National Cancer Institute, Bethesda, Maryland, United States of America; Seoul National University College of Pharmacy, REPUBLIC OF KOREA

## Abstract

The presence of cancer stem cells (CSCs) and the induction of epithelial-to-mesenchymal transition (EMT) in tumors are associated with tumor aggressiveness, metastasis, drug resistance, and poor prognosis, necessitating the development of reagents for unambiguous detection of CSC- and EMT-associated proteins in tumor specimens. To this end, we generated novel antibodies to EMT- and CSC-associated proteins, including Goosecoid, Sox9, Slug, Snail, and CD133. Importantly, unlike several widely used antibodies to CD133, the anti-CD133 antibodies we generated recognize epitopes distal to known glycosylation sites, enabling analyses that are not confounded by differences in CD133 glycosylation. For all target proteins, we selected antibodies that yielded the expected target protein molecular weights by Western analysis and the correct subcellular localization patterns by immunofluorescence microscopy assay (IFA); binding selectivity was verified by immunoprecipitation−mass spectrometry and by immunohistochemistry and IFA peptide blocking experiments. Finally, we applied these reagents to assess modulation of the respective markers of EMT and CSCs in xenograft tumor models by IFA. We observed that the constitutive presence of human hepatocyte growth factor (hHGF) in the tumor microenvironment of H596 non-small cell lung cancer tumors implanted in homozygous *hHGF* knock-in transgenic mice induced a more mesenchymal-like tumor state (relative to the epithelial-like state when implanted in control SCID mice), as evidenced by the elevated expression of EMT-associated transcription factors detected by our novel antibodies. Similarly, our new anti-CD133 antibody enabled detection and quantitation of drug-induced reductions in CD133-positive tumor cells following treatment of SUM149PT triple-negative breast cancer xenograft models with the CSC/focal adhesion kinase (FAK) inhibitor VS-6063. Thus, our novel antibodies to CSC- and EMT-associated factors exhibit sufficient sensitivity and selectivity for immunofluorescence microscopy studies of these processes in preclinical xenograft tumor specimens and the potential for application with clinical samples.

## Introduction

Phenotypic plasticity is essential for tumor proliferation and dissemination, enabling malignant cells to maintain an adherent, epithelial-like state facilitating tumor growth, as well as the ability to switch to a motile, mesenchymal-like condition supporting metastasis and the seeding of new tumors. Such an epithelial-mesenchymal transition (EMT) in tumor tissue has classically been thought to be associated with metastasis, poor prognosis, and drug resistance, though it has more recently been suggested that “hybrid” or “partial” epithelial-mesenchymal tumor cell phenotypes (rather than transition to a fully mesenchymal phenotype) may be more strongly indicative of aggressiveness and drug resistance in some tumor types [1–4]. Likewise, the phenotypic plasticity of cancer stem cells (CSCs) allows them to seed new tumors and survive therapeutic intervention to facilitate tumor regrowth. While the precise relationship between EMT and acquisition of CSC-like properties is not fully understood and may vary amongst tumors due to genetic and microenvironmental differences [5], the clinical significance of both processes warrants the development of reagents to detect and quantitate critical proteins associated with each to further characterize how EMT influences stemness, and vice versa, in tumor cells.

To address the paucity of antibody reagents enabling specific detection of known epitopes on EMT- and CSC-associated proteins, we identified several target proteins involved in these two processes for which there was an unmet need for high-quality antibody reagents within the research community. These target proteins include the EMT-/mesenchymal-associated transcription factors Goosecoid (GSC), Sox9, Slug, and Snail, as well as the CSC marker CD133 (S1 Table). While commercially available antibodies to each of these factors now exist, such antibodies often recognize unknown or proprietary epitope sequences (S2 Table). This lack of epitope information complicates antibody-based analyses because biological variables (including changes in post-translational modifications and the presence of different splice isoforms) and technical variables (e.g., the denaturation agent and incubation time used) may alter the availability of an epitope for antibody binding [6, 7]; erroneous results due to epitope availability issues are more difficult to predict and detect when the epitope is unknown. For example, epitope recognition by the classic, widely used CD133 antibodies that target unknown epitopes AC133 and AC141 is influenced by differences in glycosylation patterns and/or protein conformations, and use of these antibodies has led to false negatives in which antibody binding is lost despite ample CD133 cell surface expression [7–9].

Following development and *in vitro* validation of our novel antibodies, we employed these reagents in immunofluorescence microscopy assays (IFAs) to assess changes in EMT-associated transcription factor and CSC marker expression in xenograft models following drug treatment or tumor microenvironment modulation. In the latter case, we took advantage of the known role of HGF/Met signaling in inducing EMT [10] and compared EMT transcription factor (TF) expression in human non-small cell lung cancer (NSCLC) H596 tumors grown in severe combined immunodeficiency (SCID) mice to those implanted in SCID mice containing a homozygous human *HGF* (*hHGF*) knock-in mutation. *hHGF* knock-in H596 models exhibit rapid tumor growth and constitutive tumor EMT due to hHGF secretion by mouse stromal cells, thereby enhancing tumor HGF/MET signaling [11]; H596 tumors are particularly receptive to enhanced HGF secretion due to the presence of a *MET* exon 14−skipping mutation that suppresses proteasome-mediated MET degradation [12]. To validate the *in vivo* use of our antibody for the CSC marker CD133, we compared SUM149PT triple-negative breast cancer (TNBC) xenograft models treated with vehicle or the CSC-targeting focal adhesion kinase (FAK) inhibitor VS-6063 (defactinib; formerly PF-04554878) [13]. Together, these experiments demonstrate the fitness-for-purpose of our novel antibodies for use in preclinical studies of EMT- and CSC-associated tumor cell plasticity; we believe that the availability of these reagents could help to address important outstanding questions regarding the role of plasticity in tumor growth, metastasis, and therapeutic resistance.

## Materials and methods

### Recombinant protein cloning, expression, and purification

Plasmid clones were obtained from the DNASU plasmid repository (Arizona State University) or had been previously generated in the laboratory of Robert Weinberg, Ph.D. at the Massachusetts Institute of Technology, Cambridge, MA. Primers for target gene amplification were designed using the Express Primer Tool [14] and were obtained from Integrated DNA Technologies (IDT). Amplicons were cloned into pMCSG7 vectors to enable expression of N-terminally 6X-histidine−tagged proteins. Large-scale culturing of the His-tagged expression clones was conducted in 2-L plastic bottles using the method of C. Sanville Millard et al. [15]. Soluble proteins were isolated by a previously-described method [16]. Plasmids encoding the full-length or truncated target proteins are available through the DNASU repository (https://dnasu.org/DNASU).

Expression and purification of soluble proteins followed a general set of methods described by the Structural Genomics Consortium et. al [17]. Most of the proteins and protein domains expressed were insoluble, and an inclusion body isolation method was therefore used for purification. Pellets were resuspended in Bacterial Protein Extraction Reagent (B-PER, Thermo Fisher Scientific, Waltham, MA) and incubated for 30 minutes at room temperature, followed by a 10-minute incubation on ice, shaking at 50 RPM. Diluted B-PER Reagent (1:10 in water) was added, and the suspension was sonicated for 3 minutes (5 seconds on, 5 seconds off), followed by a 10-minute incubation at 4°C. Cell lysates were centrifuged at 4°C for 30 minutes at 34,540 x *g*, and cell pellets were decanted and washed with 1:10 B-PER two more times. After the third centrifugation, pellets were resuspended in 25 mL solubilizing buffer (8 M urea, 50 mM Tris pH 8.0). The solubilized pellets were incubated for 30 minutes at room temperature, followed by a 45°C incubation for 10 minutes and centrifugation for 45 minutes at 34,540 x *g*. The resulting supernatant, containing isolated inclusion bodies, was tested for purity on SDS-PAGE. Size-exclusion chromatography was used when further purification was required.

CD133 domain A (amino acid residues 295–328, molecular weight 3856 kD) and domain B (amino acid residues 615–643, molecular weight 3403 kD) peptides for peptide blocking experiments (S3 Table) were expressed as bovine serum albumin (BSA) conjugates and purified from inclusion bodies using Tris buffer containing 6 M urea. Peptides were dialyzed stepwise to 4 M urea, then 2 M urea, and finally phosphate-buffered saline (PBS) and then concentrated.

### Antigen design and production

Regions of interest within each protein target were selected based on predicted minimal cross-reactivity with other targets; in particular, highly conserved DNA binding domains were avoided. Short sequences with surface exposure and/or high predicted antigenicity within the initial sequences were then used to generate peptide antigens. Peptide antigens were conjugated to keyhole limpet hemocyanin (KLH) or ovalbumin (OVA) carrier protein for immunization or to BSA for screening purposes.

### Immunization and blood screening

For each target protein, two 3-month-old New Zealand white rabbits were immunized via 4–5 initial subcutaneous injections and two test bleeds per rabbit. Titers from each of the two rabbits for each target protein group were first determined by Abcam (formerly Epitomics; Burlingame, CA) using a colorimetric enzyme-linked immunosorbent assay (ELISA) against BSA-conjugated peptides or recombinant proteins. Titers were independently confirmed at National Cancer Institute (NCI) Frederick using a LI-COR platform; briefly, plates for ELISA were coated overnight at 4°C with 100 ng antigen per well in 0.1 M carbonate buffer, pH 9.6. Plates were blocked with LI-COR Odyssey Blocking buffer (LI-COR, Cat#: 927–40010), and then sample was added and incubated at 24°C for 2 hours. Goat anti-rabbit IR-680 was used as the secondary antibody, and plates were read at 700 nm on a LI-COR Odyssey scanner. For each target protein, the rabbit with the higher titer was selected for splenectomy and fusion.

### Splenectomy, fusion, and hybridoma screening

Splenocytes were isolated, fused with partner cells, and plated on 96-well plates at Abcam. Plates were incubated under standard conditions, and hybridoma supernatants were screened by standard colorimetric ELISA against BSA-conjugated peptides or proteins as described above. Clones with optical density > 0.5 were considered putatively positive and were further expanded to 24-well plates. Initial subclone screening (ELISA and Western blot) was performed at Abcam, and the 12 most promising positive hybridoma supernatants for each target were shipped from Abcam to NCI at Frederick, where additional screening was performed.

### Subcloning, subclone screening (Western and IFA), and antibody production

Subcloning was performed by Abcam using the limited cell dilution method. Subclones were screened by NCI at Frederick. Initial screening was performed using Western blotting; tumor cell lysates or recombinant proteins were separated on 4–12% SDS PAGE gels in 1X 3-(N-morpholino)propanesulfonic acid (MOPS) buffer at 150 V using the Novex system (Thermo Fisher Scientific, Waltham, MA) and then transferred to nitrocellulose membranes using the iBlot system (Thermo Fisher Scientific). Blots were blocked with LI-COR Blocking Buffer and incubated overnight with hybridoma supernatants (diluted 1:30). LI-COR goat anti-rabbit IR-680 antibody was used for detection, and plates were read at 700 nm using a LI-COR Odyssey scanner.

Subclones were also examined by immunofluorescence microscopy. Formalin-fixed, paraffin-embedded (FFPE) cell pellets were generated according to the methods described at https://ncifrederick.cancer.gov/rtp/lasp/phl/immuno. Human tissues (pancreas, colon, skin, seminoma) were purchased from Pantomics., Inc (CA). Slides were stained with the rabbit monoclonal antibodies and 4',6-diamidino-2-phenylindole (DAPI), and anti-rabbit−Alexa Fluor (AF) 488 was used as the secondary antibody. Antibodies were produced in serum-free medium and underwent Protein A−based purification conducted by Abcam. All antibody subclones described in this manuscript are available through the Developmental Studies Hybridoma Bank at the University of Iowa (http://dshb.biology.uiowa.edu/) and the NCI Clinical Proteomic Technologies for Cancer antibody portal (https://proteomics.cancer.gov/antibody-portal/).

### Immunoprecipitation-mass spectrometry analysis of antibody selectivity

Monoclonal antibodies were diluted to 50 ng/μL in 1X PBS (0.1 M phosphate, 0.15 M NaCl) with 500 μm octyl–β–glucoside (“PBS/BOG”), and test proteins or peptides were diluted to 100 ng/μL (full-length protein or domain) or 25 ng/μL (peptide) in PBS/BOG. Dynabeads Protein A magnetic bead suspension (Invitrogen Corp., Campbell, CA) was diluted 1/4 (v/v) in PBS/BOG, and 200 μL of the diluted suspension was transferred to each well of a 96-well plate. A magnetic particle concentrator was used to facilitate removal, washing, and elution steps. Antibodies (200 μL/well) were incubated with the Protein A beads at room temperature for 1 hour with gentle mixing. Beads were then washed three times, and antigen solution (200 μL/well) was added. After overnight incubation at 4°C, beads were washed three times with 200 μL PBS/BOG, followed by three washes with HPLC-grade water. Bound peptides or proteins were eluted from the beads by addition of 20 μL 0.2% trifluoroacetic acid solution in HPLC-grade water. Eluted solutions were spotted directly onto the surface of the matrix-assisted laser desorption/ionization (MALDI) target (0.5–1 μL per spot) and mixed on the plate with the appropriate matrix solution. α-Cyano-4-hydroxycinnamic acid (CHCA) or sinapinic acid (SA) MassPREP MALDI Matrix (Waters Corporation, Milford, MA) was used for peptide or protein analysis, respectively. Plates were dried at room temperature before transfer to the mass spectrometer.

Peptide analysis was performed using a Bruker Ultraflex III MALDI-TOF/TOF in reflectron mode, and data analysis was performed with FlexAnalysis software. Protein analysis was performed on an Applied Biosystems Voyager-DE Pro time-of-flight mass spectrometer equipped with a CovalX HM-1 high mass detector and operated in linear mode under positive ion conditions; data analysis was carried out using “Data Explorer” software embedded in the Voyager instrument.

### Cell lines

HMLE, HMLE-GSC, HMLE-Slug, HMLE-Snail, MCF7-RAS, MCF-RAS-Sox9 were generated and cultured as described previously [18–20]. U87, HT29, NCI-H596, and SUM149PT cell lines were obtained from American Type Culture Collection (ATCC).

### Animal ethics statement

Frederick National Laboratory is accredited by the Association for Assessment and Accreditation of Laboratory Animal Care International and follows the Public Health Service Policy for the Care and Use of Laboratory Animals. All the studies were approved by the NCI Institutional Animal Care and Use Committee (IACUC) and conducted according to an approved animal care and use committee protocol in accordance with the procedures outlined in the ‘‘Guide for Care and Use of Laboratory Animals” (National Research Council; 1996; National Academy Press; Washington, DC).

### Animal model studies

Mice were housed in sterile, filter-capped, polycarbonate cages (Allentown Caging, Allentown, NJ) maintained in a barrier facility on a 12-hour light/dark cycle and were provided sterilized food and water ad libitum. For all experiments, mice were sacrificed by IACUC-approved methods (consistent with AVMA Guidelines for Euthanasia), including induction and maintenance of isoflurane anesthesia with exsanguination or exposure to carbon dioxide for a minimum of 10 minutes followed by additional observation or cervical dislocation. All animals were monitored daily for clinical signs of distress (e.g., hunched posture, roughened haircoat, inactivity, general body condition, weight loss, inappetence, self-isolation). Animals showing signs of distress were treated with wet feed/Hydrogel^TM^ if signs were mild (weight loss less than 20%; only 1 clinical sign apparent) or humanely euthanized if signs were more than mild.

Rabbits were housed in cages and maintained on light/dark cycles with air exchanges. The rabbits were provided food and water ad libitum. All rabbit experiments were conducted under IACUC-approved protocols, and rabbits were monitored daily for clinical signs of distress (e.g., lethargy, hyper-reactivity, motor response, nutritional status, coat condition, posture, respiration, and bodily functions). Rabbits showing signs of distress were treated according to the facility veterinary program under the guidance of a veterinarian, or humanely euthanized if signs were more than mild.

For the H596 *hHGF* knock-in experiment, female SCID/NCr (“SCID”) and homozygous *hHGF* knock-in (“*hHGF*^ki/ki^”) SCID/NCr mice (*HGF*^*tm1*.*1(HGF)Aveo*^ *Prkdc*^*scid*^/J) were obtained from Jackson Laboratory for Genomic Medicine (USA). The *hHGF*^ki/ki^ mice harbor 2 copies of the “humanized” knock-in mutation (*hHGF*^ki^) that replaces exon 3–6 of the endogenous mouse *Hgf* gene with exons 2–18 of the human *HGF* gene. Animals were implanted by subcutaneous injection of NCI-H596 cells, and tumor growth was monitored (*n =* 5 animals per group). Tumors were harvested after they reached 300 mg in weight, flash-frozen in liquid nitrogen and fixed in 10% neutral buffered formalin, processed, and then sectioned for IFA analysis.

For the VS-6063 experiment, SUM149PT tumors were implanted into SCID/NOD mice and staged to 150 mg tumor weight. Animals were randomized into groups (*n* = 4 per group) before initiation of treatment using a commercial software program (Study Director, Study log Systems, Inc., South San Francisco, CA). When tumors reached a volume of approximately 200 mm^3^, animals were treated with VS-6063 (67.5 mg/kg PO, BID for 21 days). Tumors were harvested four days following the last dose, flash-frozen in liquid nitrogen, fixed in 10% neutral buffered formalin, and sectioned for IFA analysis.

### Electrochemiluminescent immunoassay measurement of plasma hHGF levels in *hHGF*^*ki/ki*^ mice

Human HGF protein content in mouse plasma was measured using a two-site electrochemiluminescent immunoassay developed for use with a Meso Scale Discovery 2400 plate reader as described previously [21]. Antibodies for HGF capture (MAB-694) and detection (AF-294) and a purified recombinant HGF protein reference standard (294-HG) were obtained from R&D Systems (Minneapolis, MN). The lower limit of detection for this assay is 0.75 pg/mL in 96-well format using a 25 μL sample volume and a total protein concentration of 0.25 mg/mL. All samples were measured in triplicate unless otherwise noted.

### Xenograft biopsy and tumor tissue microarrays (TMAs) for target peptide or recombinant protein blocking experiments

Whole-xenograft tumors were collected by standard dissection methods. Specimens were cut into two to four equal pieces with fine-point scissors or a single-edge scalpel and immediately flash frozen in a liquid nitrogen−pre-cooled, o-ring−sealed, conical-bottom, screw-capped 1.5-mL Sarstedt cryovial by touching the biopsy core to the inside of the tube within one minute of excision. Tubes with frozen tissues or cells were pre-chilled on dry ice, fixed in 10% neutral buffered formalin (Sigma-Aldrich, St. Louis, MO) for 48 hours, and embedded in paraffin before being arrayed using a 1.0-mm core needle arrayer.

### Immunofluorescence microscopy analysis of xenograft tumor specimens

FFPE tissues were cut into 5 μm sections and placed on slides. Staining was performed using a BOND-MAX Autostainer (Leica Microsystems, Wetzlar, Germany). Heat-induced antigen retrieval was performed by incubation in citrate (pH 6.0) at 100°C for 10 minutes. For EMT multiplex IFA detection, fluorescence-conjugated rabbit monoclonal antibodies to E-cadherin (clone 36, Alexa Fluor (AF) 488, BD Biosciences, San Diego, CA) and vimentin (clone V9, AF790, Santa Cruz Biotechnology, Dallas, TX) were used. EMT TF multiplex IFA was performed on adjacent tumor tissue sections using the E-cadherin antibody described above, as well as unconjugated rabbit monoclonal antibodies (Snail 41–7, Slug 9–12, Sox9 15–4, or GSC 1-5) as primary antibodies and 10 μg/mL of goat anti-rabbit AF 546 (Life Technologies, Carlsbad, CA) as the secondary antibody. CSC multiplex IFA was performed using the unconjugated rabbit monoclonal CD133 47–10 antibody and commercially available mouse monoclonal anti-CD44v6 antibody (clone 2F10, R&D Systems) as primary antibodies and 10 μg/mL of goat anti-rabbit AF546 (Life Technologies) and anti-mouse AF488, respectively, as secondary antibodies. Image acquisition was performed using Aperio FL Scanner and Spectrum Analysis software and a wide-field fluorescent and confocal microscope (Nikon 90i Andor Camera, NIS Elements Software). Image segmentation and quantitative multiplex analysis were performed using Definiens software (Tissue Studio Architect).

### Target peptide or recombinant protein blocking of antibody signal in immunohistochemistry or immunofluorescence microscopy analyses

Synthetic peptides (New England Peptide, Gardner, MA) corresponding to the target epitopes of the GSC, Sox9, and Slug antibodies (see S3 Table) were each incubated at 100-fold molar excess with 10 μg of the respective antibody overnight at 4°C. Antibodies incubated with buffer alone were used as controls. Antibodies incubated with buffer or blocking peptides were used for immunohistochemical analyses of H596 tumor TMAs from *hHGF*^ki/ki^ mice using an anti-rabbit−HRP secondary antibody. Similarly, full-length recombinant Snail protein was incubated at 10-fold molar excess with the Snail 41–7 antibody to block IFA staining on H596/*hHGF*^ki/ki^ TMA slides using an anti-rabbit−AF488 secondary antibody. Peptides corresponding to CD133 domain A (amino acid residues 295–328, molecular weight 3856 kD) and domain B (amino acids 615–643, molecular weight 3403 kD) were incubated at 20-fold molar excess with the CD133 47–10 antibody overnight at 4°C to block IFA staining of HT29 FFPE cell pellet blocks using an anti-rabbit AF546 secondary antibody.

### Flow cytometry using anti-CD133 antibodies

W6B3C1, 293C3, and AC133 antibodies were obtained from Miltenyi Biotec (Bergisch Gladbach, Germany). HT29 cells were harvested from cell culture plates using 10 mM EDTA in PBS. Aliquots of 1×10^6^ cells each were stained with either no antibody (control) or 10 μg/mL of CD133 47-10, W6B3C1, 293C3, or AC133 antibodies for 30 minutes at 4°C. Conjugated anti-Rabbit-AF488 or anti-Mouse-AF488 were used as secondary antibodies. Isotype control antibodies included Rabbit IgG Isotype Control (catalog number 10500C, ThermoFisher Scientific) as a control for CD133 47–10 and Alexa Fluor 647 Mouse IgG1, κ Isotype Control (catalog number 566011, BD Biosciences) as a control for W6B3C1, 293C3, and AC133.

### Statistical analyses

Statistical significance for comparisons of EMT and CSC marker expression was determined by nonparametric Mann-Whitney tests using the GraphPad Prism program (GraphPad Software, La Jolla, CA). A significance level (α) of 0.05 was used as the cut-off to define statistical significance.

## Results

### Monoclonal antibody production and *in vitro* and *in vivo* characterization

To address the need for highly specific monoclonal antibodies for quantitative assessment of EMT- and CSC-associated proteins, we generated antigenic peptides or full-length recombinant proteins corresponding to several relevant targets for use in rabbit immunization (Table 1 and S4 Table). Immunogenic peptides corresponding to the EMT TFs were designed to utilize unique protein regions distal to the conserved DNA binding regions; for example, in the case of Slug, we used a peptide corresponding to a sequence within a region of unknown function (residues 95–122) that is unique to Slug [22]. For generation of anti-CD133 antibodies, we selected antigenic peptides corresponding to regions within the first and second extracellular loops and distal from all known glycosylation sites, as glycosylation has been shown to interfere with the binding of several existing CD133 antibodies [7].

**Table 1:** Western blot and immunofluorescence microscopy characterization of rabbit monoclonal antibodies for EMT- and CSC-associated proteins.

Target	Antibody clone name (*CPTC clone name*)	Immunogen	Predicted molecular weight for WB (kD)	Overexpressed protein detected by WB (MW in kD)	Endogenous protein detected by WB (MW in kD)	Expected nuclear localization	Nuclear localization in relevant model *in vitro* system	Model systems for *in vitro* characterization
GSC	GSC 1–5 (*CPTC-GSC-1*)	recombinant GSC	38	yes (36)	no	yes	yes	HMLE vs. HMLE-GSC
Sox9	Sox9 15–4 (*CPTC-SOX9-1*)	aa 48–66	56	yes (56)	yes (56)	yes	yes	MCF7-RAS vs. MCF7-RAS-Sox9
Slug	Slug 9–12 (*CPTC-SNAI2-2*)	aa 101–116	30	yes (36)	no	yes	yes (+ cyto.)	HMLE vs. HMLE-Slug (dox-induced)
Snail	Snail 41–7 (*CPTC-SNAI1-1*)	aa 22–39	30	yes (36)	no	yes	yes	HMLE vs. HMLE-Snail (dox-induced)
CD133	CD133 47–10 (*CPTC-PROM1-1*)	aa 295–329	~97–133	n.d.	yes (~120)	no	no (cyto./PM)	U87 (low exp.) vs. HT29 (high exp.)

Nuclear localization was assessed by immunofluorescence microscopy using the antibody of interest followed by an anti-rabbit−AF488 secondary antibody. WB: Western blot; aa: amino acid; cyto.: cytosolic; dox: doxycycline; n.d.: not determined; PM: plasma membrane. The clone name under which each antibody has been deposited in the NCI Clinical Proteomic Technologies for Cancer (CPTC) antibody portal (https://proteomics.cancer.gov/antibody-portal/) and Developmental Studies Hybridoma Bank at the University of Iowa (http://dshb.biology.uiowa.edu/) is noted in italics.

Following immunization with the peptide or protein of interest, ELISA-based blood screening, splenectomy, and hybridoma production and screening (as outlined in S1 Fig), we selected specific antibody subclones based on ELISA and Western blot analyses using recombinant target proteins or target epitope−containing peptides and on immunofluorescence microscopy analyses of cancer cell lines. We then performed further characterization by immunofluorescence microscopy, ELISA, and Western blot analyses of target protein−overexpressing cell lysates and immunoprecipitation−mass spectrometry (IP-MS) detection of antibody binding to target epitope−containing peptides or recombinant full-length proteins and/or protein domains (S4 and S5 Tables). All of the antibodies presented here are available through the Developmental Studies Hybridoma Bank at the University of Iowa (http://dshb.biology.uiowa.edu/) and the NCI Clinical Proteomic Technologies for Cancer antibody portal https://proteomics.cancer.gov:antibody-portal).

**Fig 1:**
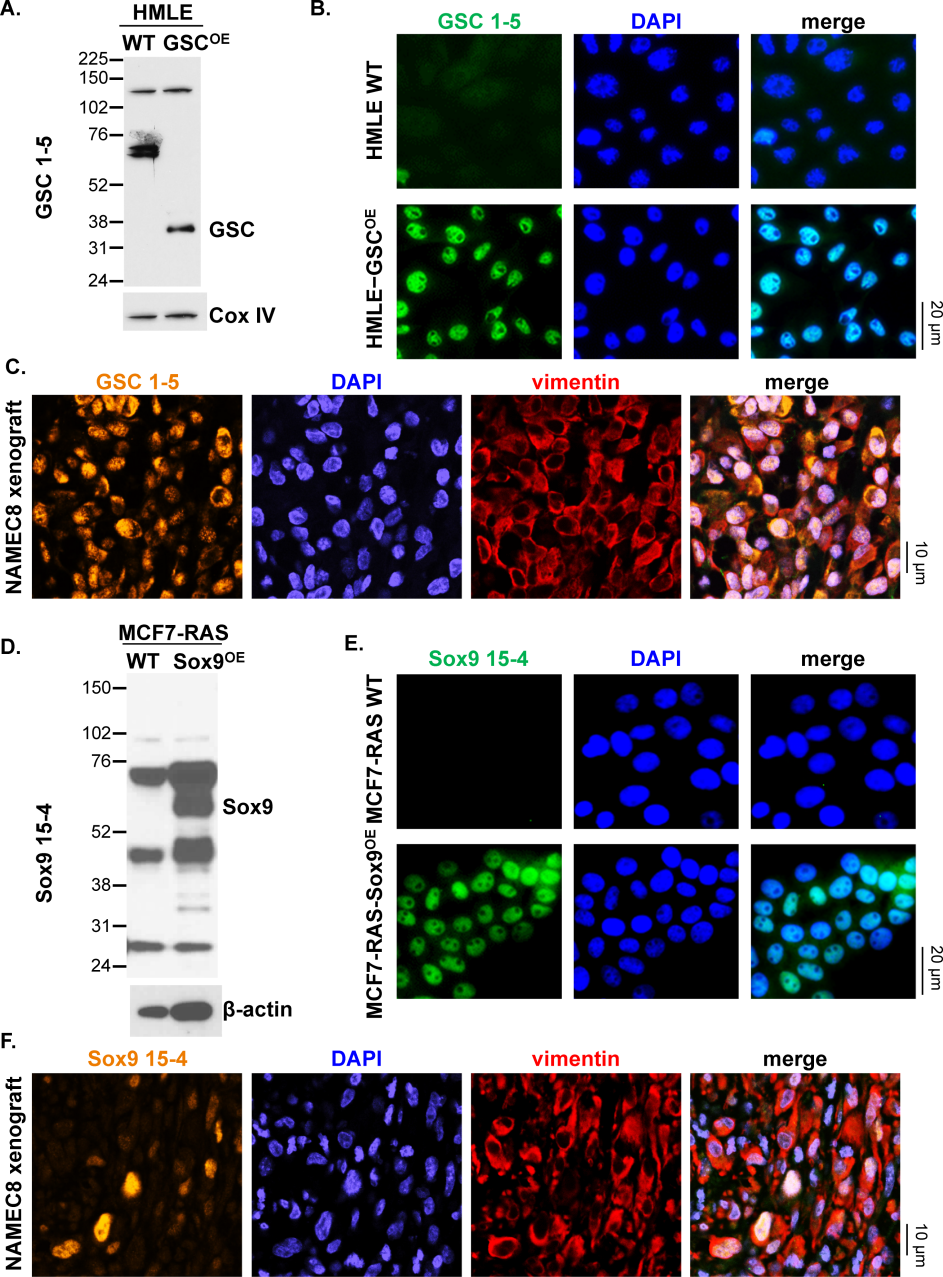
Western blot and immunofluorescence microscopy characterization of novel anti-GSC and anti-Sox9 antibodies in target protein−overexpressing cell lines and NAMEC8 xenograft models. Data are shown for GSC 1–5 **(A-C)** and Sox9 15-4 **(D-F)**. **(A**, **B**, **D** and **E)** *In vitro* target protein detection by EMT TF antibodies was assessed by comparisons of wild-type and constitutive target protein−overexpressing cell lines. **(A** and **D)** Western blot detection of GSC and Sox9, respectively. **(B** and **E)** 20X magnification immunofluorescence images show staining with DAPI (blue) and with the antibody of interest and fluorescence-conjugated anti-rabbit secondary antibody (green). **(C** and **F)** *In vivo* target protein detection by EMT TF antibodies was assessed by immunofluorescence microscopy analysis (60X magnification) of tumor tissue from NAMEC8 xenograft models (gold, EMT TF antibody; blue, DAPI; red, vimentin).

**Fig 2:**
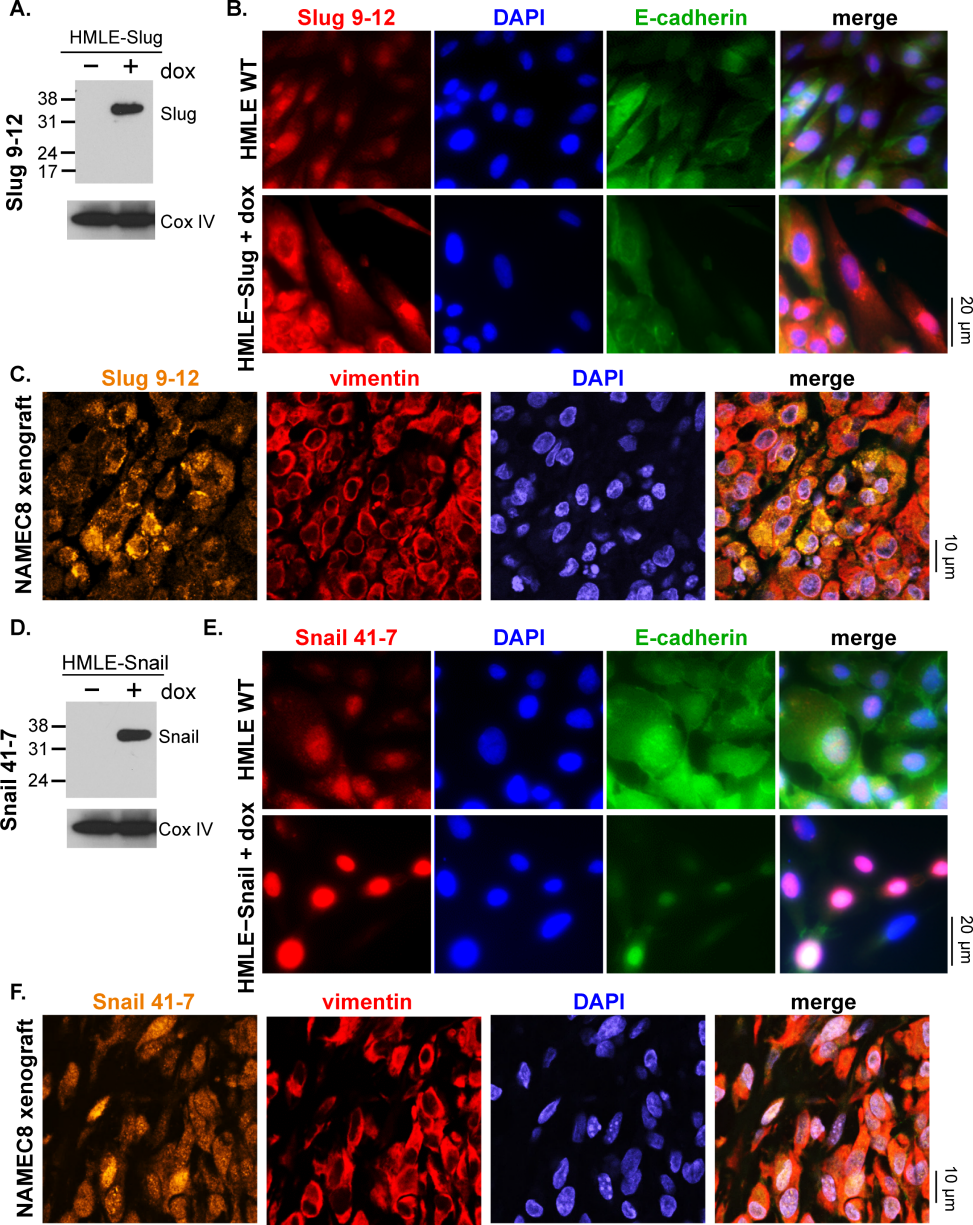
Western blot and immunofluorescence microscopy characterization of novel anti-Slug and anti-Snail antibodies in target protein−overexpressing cell lines and NAMEC8 xenograft models. Data are shown for Slug 9–12 **(A-C)** and Snail 41–7 **(D-F)**. **(A**, **B**, **D** and **E)** *In vitro* target protein detection by EMT TF antibodies was assessed by comparisons of wild-type and doxycycline (dox)-inducible target protein−overexpressing cell lines. **(A** and **D)** Western blot detection of Slug and Snail, respectively. **(B** and **E)** 20X magnification immunofluorescence images of cultured cells show staining with DAPI (blue), the antibody of interest and fluorescence-conjugated anti-rabbit secondary antibody (red), and fluorescence-conjugated anti–E-cadherin antibody (green). **(C** and **F)** *In vivo* target protein detection by EMT TF antibodies was assessed by immunofluorescence microscopy analysis (60X magnification) of tumor tissue from NAMEC8 xenograft models (gold, EMT TF antibody; blue, DAPI; red, vimentin).

**Fig 3:**
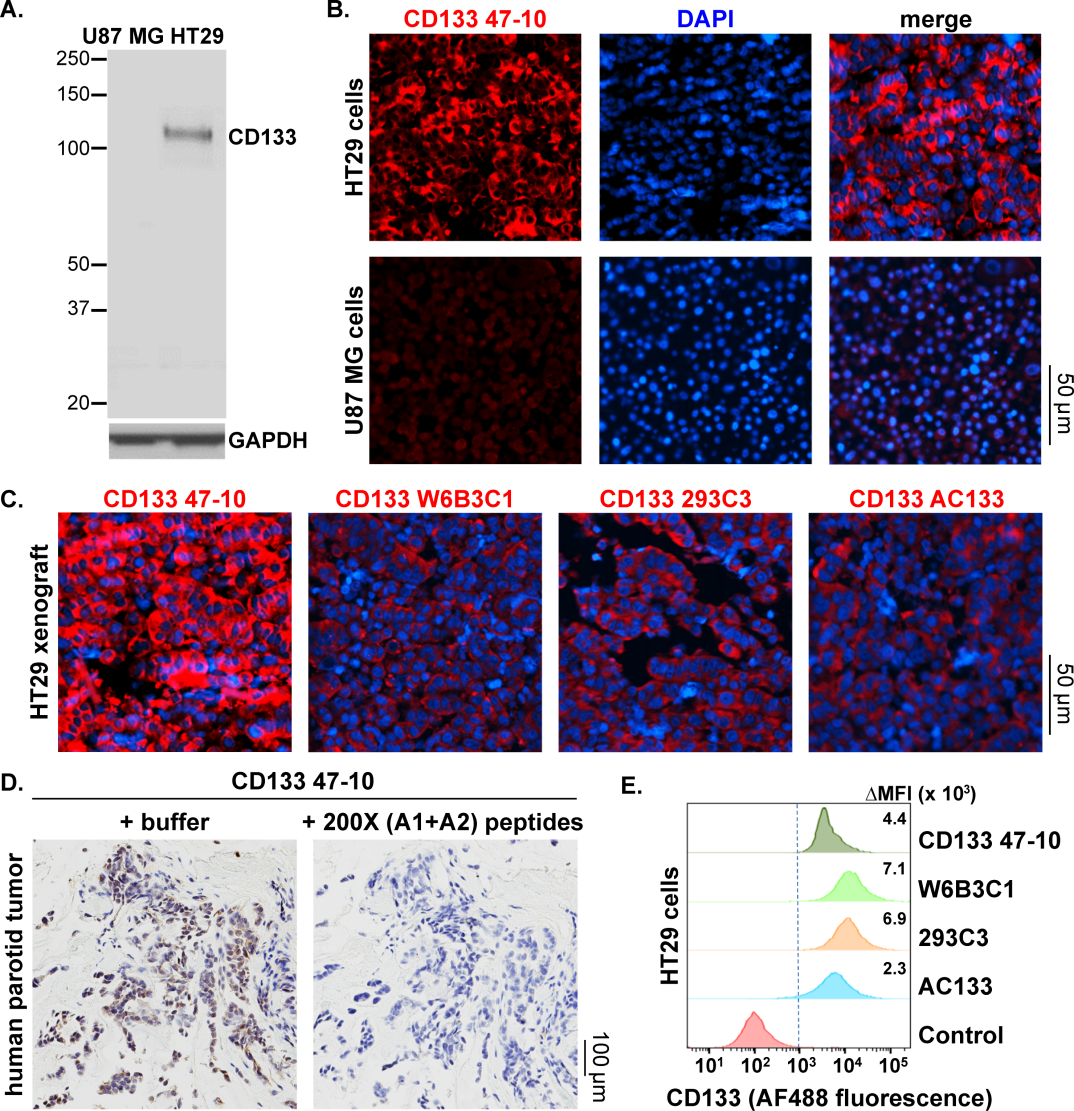
Western blot, immunofluorescence microscopy, and flow cytometry characterization of a novel anti-CD133 antibody in cell line, xenograft, and clinical tumor specimens. CD133 detection was assessed *in vitro* by comparing cell lines with known high and low endogenous CD133 expression (HT29 and U87 MG, respectively). **(A)** Western blot detection of CD133 by the CD133 47–10 antibody. **(B)** Immunofluorescence microscopy images of HT29 (top) and U87 MG (bottom) cells show staining with DAPI (blue) and CD133 47–10 and fluorescence-conjugated anti-rabbit secondary antibody (red) at 40X magnification. **(C)** Immunofluorescence microscopy images of tumor tissue from HT29 xenograft models, stained with DAPI (blue) and novel or commercially available anti-CD133 antibodies and fluorescence-conjugated anti-rabbit secondary antibody (red) at 40X magnification. W6B3C1, AC133, and 293C3 antibodies were obtained from Miltenyi Biotec and target unknown epitopes 1 (W6B3C1 and AC133) or 2 (293C3) (see S2 Table). Identical primary antibody concentrations (10 μg/mL) were used for each experiment. **(D)** Immunohistochemical analysis of CD133 expression in human parotid tumor tissue using the CD133 47–10 antibody. Tumor tissue was combined with CD133 47–10 and incubated with either buffer (left) or the “A1” and “A2” peptides (right) corresponding to portions of the domain A epitope sequence recognized by CD133 47–10, each at a concentration 200-fold higher that of the antibody (see S3 Table for blocking peptide sequences). CD133 was visualized using an HRP-conjugated anti-rabbit antibody. **(E)** Flow cytometry analysis of CD133-expressing HT29 cells using novel and commercially available anti-CD133 antibodies. “Control” indicates unstained cells; ΔMFI values, indicating the difference in median fluorescence intensities between each antibody and its matched isotype control (rabbit for CD133 47–10 and mouse for all others), are shown for each antibody.

Based on stringent selection criteria for target specificity and functional utility using these various assay platforms, we were able to validate antibodies to GSC, Sox9, Slug, Snail, and CD133 (Table 1, Table 2 and S4 Table). Antibodies were tested for their ability to detect target protein overexpression by Western blot and to detect both target protein expression and proper intracellular localization by immunofluorescence microscopy using various relevant cell line and xenograft models (Table 1 and Figs 1–3); in both types of assays, unlabeled primary antibodies were used prior to application of a fluorescence-labeled anti-rabbit secondary antibody. Anti−EMT TF antibodies were tested in parental and matched EMT protein−overexpressing cell lines, including non-conditional (GSC) or doxycycline-inducible (Slug and Snail) overexpression in SV40− and human telomerase reverse transcriptase (hTERT)−immortalized human mammary epithelial cells (HMLEs) [19]. Similarly, anti-Sox9 antibodies were assessed in transformed, MCF7-RAS cells with or without overexpression of Sox9 [20, 23]. Antibodies to CD133 were tested in U87 MG and HT29 cells, which are known to express low and high levels, respectively, of endogenous CD133 [24]; HT29 cells also express high levels of other known CSC markers, such as Nanog, ALDH1, and pY397-FAK, compared to other tumor cell lines (S2 Fig). The anti−EMT TF antibodies successfully detected by Western blot the higher expression of the corresponding target protein in target protein−overexpressing cells compared to wild-type cells and yielded the expected nuclear staining pattern by fluorescence microscopy analysis of both target protein−overexpressing cells and tumor tissue from the Naturally Arising MEsenchymal Cell 8 (NAMEC8) xenograft model [18] (Figs 1 and 2). In the case of the anti-Slug antibody, both nuclear and cytoplasmic staining were observed in doxycycline-induced Slug-overexpressing HMLE cells and NAMEC8 xenograft tumor tissue (Fig 2B and 2C), as has been observed previously in other tumor cell lines and tissue specimens [25–28]. The anti-CD133 antibody successfully detected CD133 in HT29 but not U87 MG cells and produced the expected plasma membrane staining pattern in HT29 cells and xenograft tumor tissue (Fig 3A–3C).

**Table 2:** IP-MS characterization of target binding selectivity for antibodies to EMT- and CSC-associated proteins.

Antibody clone name	Epitope residue numbers	Binding to peptide		Binding to protein domain	
		epitope	non-epitope	epitope	non-epitope
GSC 1–5	2–18	**y** (aa 2–18)	**n** (aa 74–93)	**y** (full-length)	**n.d.**
Sox9 15–4	48–66	**y** (aa 48–66)	**n** (aa 437–455)	**y** (aa 1–150)	**n** (aa 375–509)
Slug 9–12	101–116	**y** (aa 101–116)	**n** (aa 90–104)	**y** (full-length)	**n.d.**
Snail 41–7	22–39	**y** (aa 22–39)	**n.d.**	**y** (full-length)	**n.d.**
CD133 47–10	295–329	**n.d.**	**n.d.**	**y** (aa 180–400)	**n** (aa 515–745)

Selectivity for the indicated epitopes was determined by IP-MS using the antibody of interest and corresponding epitope-containing (“epitope”) or non-epitope-containing (“non-epitope”) peptides and/or recombinant full-length protein or protein domains, as noted. Binding (yes/no) and sequence residue numbers for epitope and non-epitope peptides and protein domains (in parentheses) are shown.

aa: amino acid; n: no; n.d.: not determined; y: yes

To ensure selectivity of the chosen antibodies in preparation for IFA analyses of tumor tissue, we performed IP-MS analyses of epitope- and non-epitope-containing peptides and/or protein domains, as well as peptide blocking IFA or immunohistochemistry experiments using target protein−expressing cell lines or xenograft or human tumor tissues. IP-MS results confirmed that the anti-Sox9, -Slug, -Snail, and -CD133 antibodies bound selectively to the epitope-containing peptides and proteins, with no binding detected for the non-epitope-containing molecules tested (Table 2). In the case of the GSC antibody, which was generated using full-length, recombinant GSC as an immunogen, IP-MS experiments with GSC peptides identified the epitope-containing sequence (amino acid residues 2–18). The selectivity of each antibody was further demonstrated in peptide blocking microscopy experiments, which revealed that the strong immunohistochemical/immunofluorescence signal observed in cell pellets or xenograft tissue specimens was eliminated when the antibody of interest and corresponding cells or tissue were incubated with molar excess of unlabeled peptide or protein containing the target epitope (Fig 3D, S3 Fig and S3 Table). In the case of CD133 47–10, the immunofluorescence and immunohistochemical signals in HT29 cell pellets and human parotid tumor tissue, respectively, were blocked by peptides corresponding to the epitope-containing region in the first extracellular loop (“domain A”; Fig 3D and S3 Fig); however, the CD133 47-10 immunofluorescence signal in HT29 cells was not blocked by a peptide corresponding to the second extracellular loop (“domain B”), further demonstrating the selectivity of this reagent (S3 Fig). Together, these IP-MS and microscopy results confirm the selectivity of the novel EMT and CD133 antibodies for microscopy studies.

### Comparison of novel and commercially available anti-CD133 antibodies in immunofluorescence microscopy and flow cytometry applications

To further illustrate the value of our novel CD133 47–10 antibody for assessing CD133 levels in tumor cells and tissue, we compared the performance of this antibody, in immunofluorescence microscopy and flow cytometry applications, to 3 commercially available anti-CD133 monoclonal antibodies that have been used for analysis of clinical specimens: clones W6B3C1, 293C3, and AC133 from Miltenyi Biotec [7, 29, 30]. These antibodies target unknown epitopes, referred to as “epitope 1” (W6B3C1 and AC133) and “epitope 2” (293C3; see S2 Table), that are thought to be altered by changes in glycosylation and/or protein conformations [7–9]. CD133 47–10 outperformed these commercial antibodies in immunofluorescence microscopy experiments, yielding a stronger fluorescence signal relative to the latter reagents when used at identical concentrations (Fig 3C). We also examined the suitability of CD133 47–10 for flow cytometry analysis of CD133-expressing HT29 cells, again comparing this antibody to the commercially available W6B3C1, 293C3, and AC133 antibodies. While CD133 47–10 successfully distinguished CD133-positive cells by flow cytometry, the intensity of the fluorescence signal was slightly lower for CD133 47–10 compared to the commercial antibodies in this experiment (Fig 3E); the median difference in fluorescence intensity (ΔMFI) between each antibody and its matched isotype control was highest for W6B3C1 (7.1×10^3^), followed by 293C3 (6.9×10^3^), CD133 47–10 (4.4×10^3^), and then AC133 (2.3×10^3^). Together, these immunofluorescence microscopy and flow cytometry studies demonstrate the ability of the CD133 47–10 antibody to detect CD133-positive cells in these two applications in a manner that is comparable or superior to that of commonly used commercially available anti-CD133 antibodies.

### Application of novel antibodies to characterize EMT in human HGF knock-in non-small cell lung cancer mouse xenograft models

**Fig 4:**
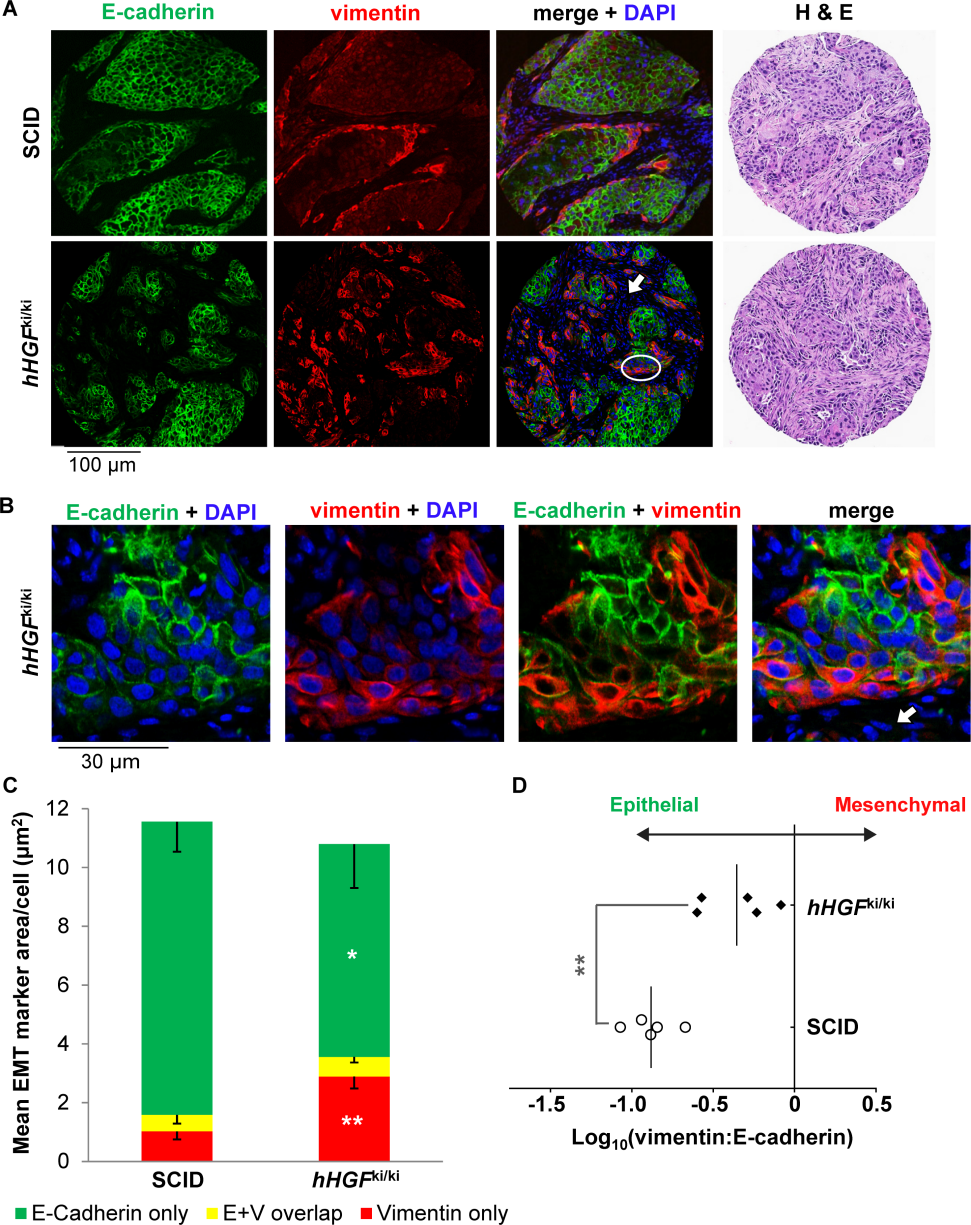
NSCLC tumors from xenograft models with constitutive plasma and stromal hHGF expression exhibit enhanced mesenchymal-like character. SCID or homozygous *hHGF* knock-in (*hHGF*^ki/ki^) mice were implanted with H596 NSCLC tumors (*n* = 5 animals per group), and tumors were harvested 33 days after implantation. **(A)** Representative EMT TF immunofluorescence microscopy images of H596 tumors from SCID or *hHGF* knock-in animals (20X magnification). FFPE tissue sections were assessed by immunofluorescence microscopy after staining with DAPI (blue) or fluorescence-conjugated primary antibodies to E-cadherin (green) and vimentin (red) or by light microscopy following H & E staining (far right). White arrow indicates a representative region rich in hHGF-secreting mouse stromal cells, which are not recognized by the anti−human E-cadherin or vimentin antibodies. White circle indicates the region shown at higher magnification in panel B. **(B)** High-magnification (60X) images of the circled region in panel A, with arrow indicating representative hHGF-secreting mouse stromal cells. **(C)** Quantitation of E-cadherin-only, vimentin-only, and E-cadherin + vimentin colocalized (E+V) staining in H596 tumors harvested from SCID or *hHGF* knock-in animals. Error bars represent standard deviation, and significant changes in the mean marker area per cell between the two groups are indicated (*P < 0.05; **P < 0.01). **(D)** Log_10_(vimentin:E-cadherin) values for H596 tumors from SCID and *hHGF* knock-in animals reveal shifts toward a more mesenchymal-like phenotype for tumors exposed to constitutive stromal hHGF. Each point represents an individual animal; the significant difference between log_10_(vimentin:E-cadherin) values for the two groups is indicated (**P < 0.01).

To illustrate the value of our novel EMT TF antibodies for examining tumor cell EMT in a complex histological context, we used these antibodies to assess EMT TF expression in a human *HGF* (*hHGF*) knock-in mouse xenograft model in which implanted human H596 non-small cell lung cancer (NSCLC) tumors undergo induction of EMT due to: 1) constitutive expression and secretion of hHGF by mouse stromal cells in the tumor microenvironment [11] and 2) potentiated activity of the human MET receptor in H596 tumors resulting from a *MET* exon 14−skipping mutation that suppresses MET proteasomal degradation [12]. Relative to H596 NSCLC tumors implanted in wild-type SCID mice, H596 tumors implanted in homozygous human *HGF* knock-in (*hHGF*^ki/ki^) mice exhibit enhanced growth rates and mesenchymal-like phenotypes, the latter assessed via immunofluorescence assays for E-cadherin and vimentin, markers of the epithelial and mesenchymal states, respectively [11]. We first confirmed that H596 tumor−bearing *hHGF*^ki/ki^ mice exhibited substantially higher plasma hHGF levels compared to H596 tumor−bearing SCID mice, which had plasma hHGF levels below the lower limit of quantitation (S4 Fig). This enhanced plasma hHGF was associated with significant downregulation of E-cadherin (*P* < 0.05), upregulation of vimentin (*P* < 0.01), and, hence, increased mesenchymal-like character (as measured by the log_10_ vimentin:E-cadherin ratio; *P* < 0.01) of H596 tumors isolated from *hHGF*^ki/ki^ mice (Fig 4).

**Fig 5:**
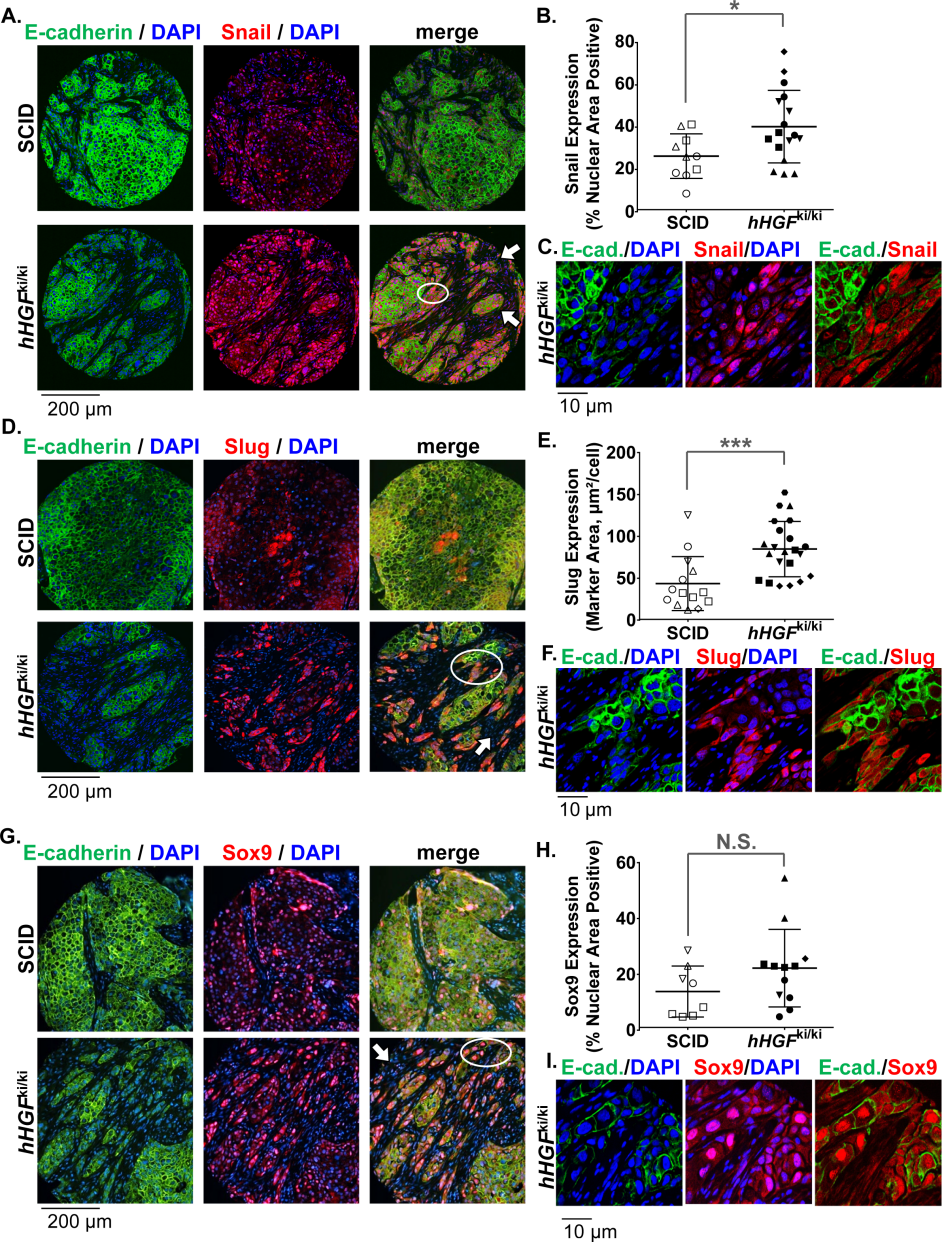
Novel antibodies detect upregulation of EMT transcription factor expression coinciding with reduced E-cadherin expression in a NSCLC xenograft model with constitutive HGF signaling. SCID or homozygous *hHGF* knock-in mice were implanted with H596 NSCLC tumors, and tumors were harvested 33 days after implantation. FFPE tissue sections were assessed by immunofluorescence microscopy after staining with DAPI (blue) or with fluorescence-conjugated anti-rabbit antibodies following application of primary antibodies to E-cadherin (green) and EMT TFs (red), including Snail **(A-C)**, Slug **(D-F)**, and Sox9 **(G-I)**. **(A, D, G)** Representative 20X magnification images for each group (SCID or *hHGF* knock-in). White arrows indicate examples of regions rich in hHGF-secreting mouse stromal cells, which are not recognized by the anti−human E-cadherin, Sox9, Slug, or Snail antibodies. White circles indicate the regions shown at higher magnification in panels **(C)**, **(F)**, and **(I)**, respectively. **(B, E, H)** Quantitation of tumor cell nuclear area positive for Snail **(B)** and Sox9 **(H)** and tumor area positive for Slug **(E)** was performed using Definiens software. Means ± standard deviations are shown (black bars). Each point represents the EMT TF expression value for a single tumor core; points with the same symbol indicate cores harvested from the same animal. Significant differences in EMT TF expression between SCID and homozygous *hHGF* knock-in mice are indicated (*P < 0.05, ***P < 0.001, N.S.: not significant; *n* = 3–6 animals per group). **(C, F, I)** High-magnification (60X) images of the circled regions in panels **(A)**, **(D)**, and **(G)**, respectively.

Application of our novel antibodies revealed that in H596 tumors from SCID mice, expression of Sox9, Slug, and Snail was relatively low, largely overlapped with E-cadherin expression, and localized primarily to tumor cells exhibiting a cobblestone-like epithelial morphology (Fig 5). In contrast, H596 tumors from *hHGF*^ki/ki^ mice exhibited generally increased expression of these EMT TFs, particularly in spindle-shaped, morphologically mesenchymal cells lacking E-cadherin expression. These EMT TF−positive, morphologically mesenchymal cells were observed in small, isolated clusters within the stroma or at the tumor-stroma interface (Fig 5), as would be expected given the role of hHGF secreted by mouse stromal cells in activating tumor cell EMT. Quantitation of EMT TF expression using Definiens software revealed statistically significant increases in Snail and Slug expression in H596 tumors from *hHGF*^ki/ki^ relative to those from SCID mice (*P* < 0.05 and *P* < 0.001, respectively), while the slight increase in Sox9 expression was not statistically significant (Fig 5B, 5E and 5H). In addition, no substantial changes in GSC expression patterns were observed in H596 tumors from *hHGF*^ki/ki^ versus SCID mice (data not shown), which might be explained by the independent regulation of GSC relative to other EMT TFs [19]. Thus, our novel antibodies to EMT transcription factors enabled detection of the expected increase in mesenchymal-like character of H596 tumors in *hHGF*^ki/ki^ compared to SCID mice and delineation of the specific EMT TFs associated with this mesenchymal-like state.

### Application of novel anti-CD133 antibody to assess CSC phenotype modulation following treatment of triple-negative breast cancer xenograft models with the FAK/CSC inhibitor VS-6063

**Fig 6:**
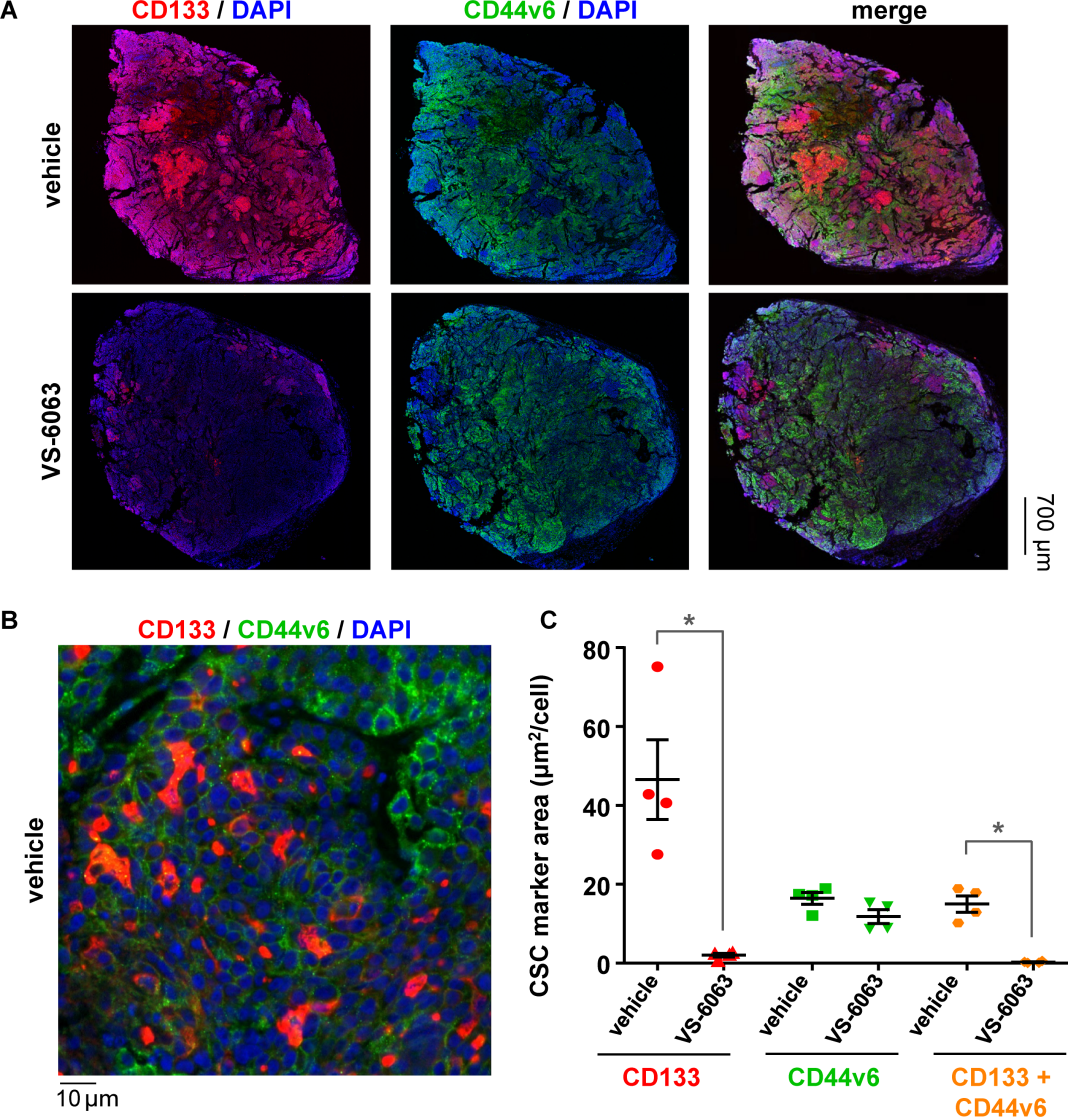
Novel anti-CD133 antibody detects reduction in CD133-positive tumor area following FAK/CSC inhibitor VS-6063 treatment in the SUM149PT triple-negative breast cancer xenograft model. Mice bearing SUM149PT xenografts were treated with vehicle or the FAK and putative cancer stem cell inhibitor VS-6063 (67.5 mg/kg PO, BID) for 21 days. Tumors were harvested four days following the last dose, fixed in 10% neutral buffered formalin, and sectioned. **(A)** Representative 20X magnification images of vehicle- (top) and VS-6063-treated (bottom) tumor sections stained with DAPI (blue), our novel CD133 47–10 antibody and corresponding fluorescence-conjugated secondary antibody (red), and a commercially available CD44v6 antibody (R&D Systems, clone 2F10) and corresponding fluorescence-conjugated secondary antibody (green). **(B)** High-magnification (100X) image of vehicle-treated tumor section from (A), with colors as indicated above. **(C)** Quantitation of tumor area positive for total CD133 (red), total CD44v6 (green), and CD133/CD44v6 colocalization (orange) was performed using Definiens software. Means ± standard errors of the mean are shown. Significant differences in CSC marker−positive area between vehicle and VS-6063−treated groups are indicated (*P < 0.05; *n* = 4 animals per group).

To demonstrate the utility of our novel anti-CD133 antibody for assessing modulation in tumor CSC phenotype, we applied CD133 47–10 to tumor tissue sections from xenograft models treated with the FAK inhibitor VS-6063 (defactinib; formerly PF-04554878), which preferentially targets CSCs and has been shown to reduce metastatic outgrowth in TNBC xenograft models [13]. For this experiment, we selected the SUM149PT TNBC xenograft model, as the corresponding cell line is known to contain populations of both differentiated and CSC-like, undifferentiated cells [31]. Mice harboring SUM149PT tumors were treated with vehicle or 67.5 mg/kg VS-6063 twice daily for 21 days, and tumors were harvested four days following the last dose and sectioned for immunofluorescence microscopy and quantitative analysis using Definiens software (Fig 6). As expected, we observed a significantly smaller mean CD133-positive tumor cell area for the VS-6063−treated group relative to the vehicle group (2.1 vs. 46.6 μm^2^/cell, respectively; *P <* 0.05) (Fig 6C). To further explore this drug-induced change in CSC character, we employed a commercially available antibody to assess levels of CD44v6, a CSC-associated isoform of CD44 that promotes tumor cell motility and is correlated with poor prognosis in breast cancer patients [32–34]. The CD44v6-positive tumor cell area was slightly (but not significantly) lower for VS-6063−treated tumors relative to the vehicle group (Fig 6A and 6C), suggesting that our novel anti-CD133 antibody may allow for more sensitive detection of CSC modulation in the SUM149PT model.

## Discussion

We have demonstrated the affinity, selectivity, and preclinical fitness-for-purpose of several antibodies for the detection and quantitation of proteins associated with epithelial-to-mesenchymal transition and cancer cell stemness. Our characterization of the epitope sequence for each of the presented antibodies renders these reagents particularly useful in identifying bona fide differences in protein expression, in contrast to several existing antibodies to EMT and CSC factors for which the target epitopes are unknown or proprietary. For example, in the case of ubiquitously used CD133 antibodies targeting the unknown epitopes AC133 and AC141 (both thought to be located in the second extracellular loop), the dependence of epitope recognition on specific glycosylation patterns and/or protein conformations has led to erroneous results [7–9]. Because our novel anti-CD133 antibody, CD133 47-10, was designed to target an epitope distal to sites of glycosylation, this antibody may offer improved accuracy for CD133 detection. While not all antibody targets are confounded by such severe epitope masking issues, the identification of epitopes for all of the novel antibodies presented here may enable more precise analysis of protein expression relative to the many existing commercial antibodies targeting unknown or undisclosed epitopes. For example, we found that CD133 47–10 yielded a stronger fluorescence signal by IFA relative to other commercially available antibodies targeting AC133 and AC141.

Our application of the EMT TF antibodies in xenograft experiments with the human HGF knock-in model illustrates the importance of examining multiple EMT-associated factors when characterizing tumor EMT phenotype. Quantitation of tumor vimentin and E-cadherin in the H596-*hHGF*^ki/ki^ model represents the first definitive demonstration of EMT in these tumors, and our findings are in agreement with previous *in vivo* studies of small cell lung cancer xenograft models demonstrating an association between HGF/Met pathway activation and increased expression of mesenchymal markers and tumorigenic and chemoresistant tumor phenotypes [35]. Our quantitation of vimentin and E-cadherin in the H596-*hHGF*^ki/ki^ model also validated the use of this model for assessment of EMT TF expression and other future studies of EMT; we found that the constitutively induced EMT in this model enabled detection of EMT TFs, which are often only transiently expressed during drug-induced EMT. Interestingly, despite the constitutive EMT in this model, as evidenced by enhanced expression of vimentin, Slug, Snail, and Sox9 (though not significantly so in the latter case), GSC expression was not elevated in H596 tumors from *hHGF*^ki/ki^ versus SCID mice. GSC regulation has been observed to differ from the regulation of other EMT TFs [19], and our data highlight the value of examining multiple EMT-associated factors in a single tissue specimen to fully characterize the dynamics of the transition or any stable hybrid EMT states.

Likewise, the domain-specific anti-CD133 antibody we developed should prove useful in non-ambiguous detection of this marker in future studies examining CSC phenotypes by immunofluorescence microscopy and flow cytometry. As in the case of EMT studies, the complexity of CSC characterization warrants assessment of multiple CSC-associated factors. Our data for VS-6063−treated SUM149 PT xenografts illustrate this point; while our CD133 47–10 antibody demonstrated a large, statistically significant decrease in CD133 following treatment with this FAK inhibitor, use of a commercial antibody to CD44v6 revealed only a small, non-significant treatment-induced decrease in this CSC factor. Though, generally speaking, discrepancies between CSC marker responses could be due to the well-documented differences in expression of and association with prognosis for various CSC markers in different tumor types [36], previous studies have identified CD44v6 as a cell motility−inducing factor in the MDA-MB-468 TNBC breast cancer cell line [34] and a prognostic factor in breast cancer patients [32, 33]. Our data indicate that, for the SUM149PT model, detection of CD133 with our novel antibody may be a more sensitive marker of CSC response to VS-6063 compared to detection of CD44v6.

The clinical importance of monitoring tumor EMT and stemness is becoming increasingly clear as evidence of the association of these features with metastasis and drug resistance continues to accumulate [4, 37]. Therefore, additional preclinical work to explore the molecular processes underlying these disease outcomes is necessary, and the antibodies presented here will aid in such endeavors. In particular, because tumor cells with “hybrid” or “partial” EMT phenotypes may be the true drivers of drug resistance and metastasis in some cancers [1–4], and given the temporal and spatial heterogeneity of EMT and CSC protein expression patterns in different histological contexts [3, 38–43], examination of multiple EMT and CSC factors within each tissue specimen may be necessary to fully understand the molecular basis of drug-resistant and metastatic tumor phenotypes. Our novel anti-EMT TF and anti-CSC antibodies are well-suited for IFA analysis of tumor tissue, and multiplex assays using several of these antibodies simultaneously will allow for careful dissection of the role of each target protein in preclinical specimens.

## Supporting information

S1 Fig Workflow for development and characterization of EMT TF and CSC antibodies. For a detailed explanation of each step, see Materials and Methods.
(PDF)

S2 Fig CSC marker expression across select *in vitro* cell lines. Cell lines (left) were incubated with primary antibodies specific to the indicated CSC-associated proteins (top), followed by the respective fluorescence-conjugated secondary antibodies, to characterize the CSC marker expression in each line (green, CD44v6; red, Nanog; gold, ALDH1; green, pY397-FAK; blue, DAPI; representative 20X images are shown). HT29, which was selected for testing CD133 antibodies due to its high CD133 expression, also exhibits high expression of other CSC markers (Nanog, ALDH1, and pY397-FAK). Primary antibodies used for this experiment include mouse monoclonal anti-CD44v6 (clone 2F10, R&D Systems, Minneapolis, MN), rabbit monoclonal anti-ALDH1 (clone EP1933Y, Abcam, Burlingame, CA), goat polyclonal anti-Nanog (catalog number AF1997, R&D Systems), and rabbit monoclonal anti-phospho-Tyr397-FAK (clone D20B1, Cell Signaling Technology, Danvers, MA).
(PDF)

S3 Fig Blocking experiments demonstrate specificity of selected novel antibodies for immunofluorescence microscopy or immunohistochemistry detection of EMT transcription factors and CSC factors. Tumor tissue from homozygous *hHGF* knock-in mice implanted with H596 NSCLC tumors (A-D) or HT29 cell pellets (E) were combined with antibodies to GSC (A), Sox9 (B), Slug (C), Snail (D), or CD133 domain A (E). In addition, the tissues/cell pellets and antibodies were incubated with either buffer/BSA (left) or peptides/protein corresponding to the target epitope of each antibody (right) at the indicated concentrations relative to that of the antibody (see S3 Table for blocking peptide/protein sequences). Target protein was visualized by immunohistochemistry using HRP-conjugated anti-rabbit antibody (A-C) or by immunofluorescence microscopy using a fluorescence-conjugated anti-rabbit secondary antibody (D-E); representative 20X images are shown.
(PDF)

S4 Fig Increased plasma human HGF levels in homozygous *hHGF* knock-in mice. SCID or homozygous *hHGF* knock-in (*hHGF*^ki/ki^) mice were implanted with H596 NSCLC tumors (*n* = 5 animals per group), and tumors were harvested 33 days after implantation. (A) Homozygous *hHGF* knock-in enhances plasma hHGF levels in H596 tumor−bearing SCID mice. SCID animals exhibited plasma hHGF levels below the lower limit of quantitation (LLQ) of 0.00075 ng/mL (red dashed line); mean plasma hHGF ± standard deviation for *hHGF*^*ki/ki*^ animals is shown (*n* = 5).
(PDF)

S1 Table EMT and CSC-associated target protein functions and associated malignancies. (DOCX)

S2 Table Epitope information for commonly used, commercially available antibodies to EMT TF and CSC proteins. The novel monoclonal antibodies presented in this manuscript are shown in bold for comparison. aa: amino acid; m: monoclonal; n: no; n.a.: no information available on company website; p: polyclonal; y: yes
*precise epitope sequence unknown or undisclosed by the company
**This column indicates whether antibody has been validated for immunofluorescence microscopy, per company product sheet.
^†^epitope may contain a glycosylation site
^‡^not validated for use on formalin-fixed or paraffin-embedded tissue, per company product sheet.
(DOCX)

S3 Table Peptides and proteins for blocking experiments with antibodies to EMT- and CSC-associated proteins. (DOCX)

S4 Table EMT and CSC antibody clone characterization summary. Results shown represent the most stringent outcome from experiments performed by 1–3 independent laboratories (aa: amino acid; L: lysate from target protein-overexpressing cells; n: no; n.d.: not determined; R: purified recombinant target protein; Rec.: recombinant; rGSC: full-length recombinant GSC; y: yes). Asterisks indicate antibodies that were selected and validated for preclinical use.
^1^All antibodies have been deposited under these names in the NCI Clinical Proteomic Technologies for Cancer (CPTC) antibody portal (https://proteomics.cancer.gov/antibody-portal/) and Developmental Studies Hybridoma Bank (DSHB; http://dshb.biology.uiowa.edu/).
^2^The recombinant protein tested was an isolated domain (see S4 Table).
^3^A nonspecific band was also observed by Western blot.
^4^"y" indicates antibodies for which sufficient staining and proper target protein subcellular localization were observed by IFA.
^5^Protein was detected in nucleus and cytoplasm by IFA.
^6^For antibodies generated using peptide immunogens, the immunogenic peptide was used for IP-MS. For antibodies generated using rGSC as an immunogen, a peptide containing aa 2-18 was used for IP-MS.
(DOCX)

S5 Table Recombinant protein domain sequences for antibody selection and validation experiments. Plasmids encoding these truncated target proteins are available through the DNASU repository https://dnasu.org/DNASU.
(DOCX)
