# Supplementary figures and images for: Novel antibody reagents for characterization of drug- and tumor microenvironment-induced changes in epithelial-mesenchymal transition and cancer stem cells
Source: PLoS One. 2018 Jun 21;13(6):e0199361. doi: 10.1371/journal.pone.0199361 (PMC6013203; doi:10.1371/journal.pone.0199361)

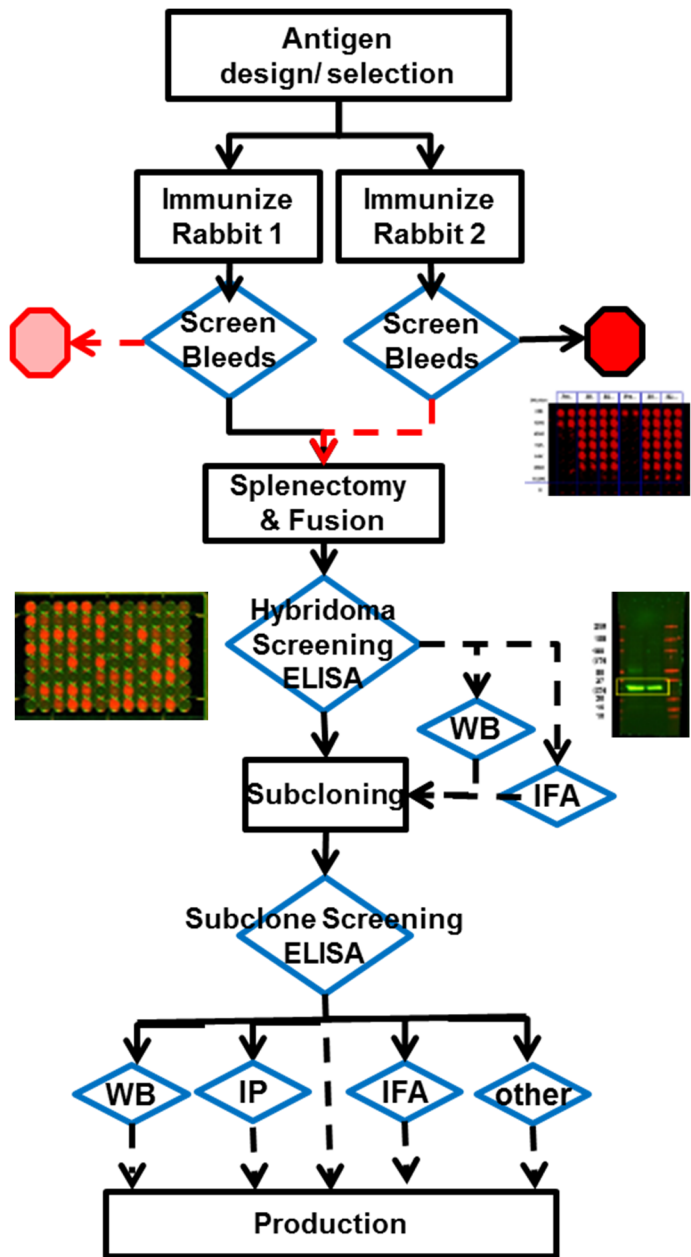

Supplement: S1 Fig — For a detailed explanation of each step, see Materials and Methods. (PDF) [file pone.0199361.s001.pdf]

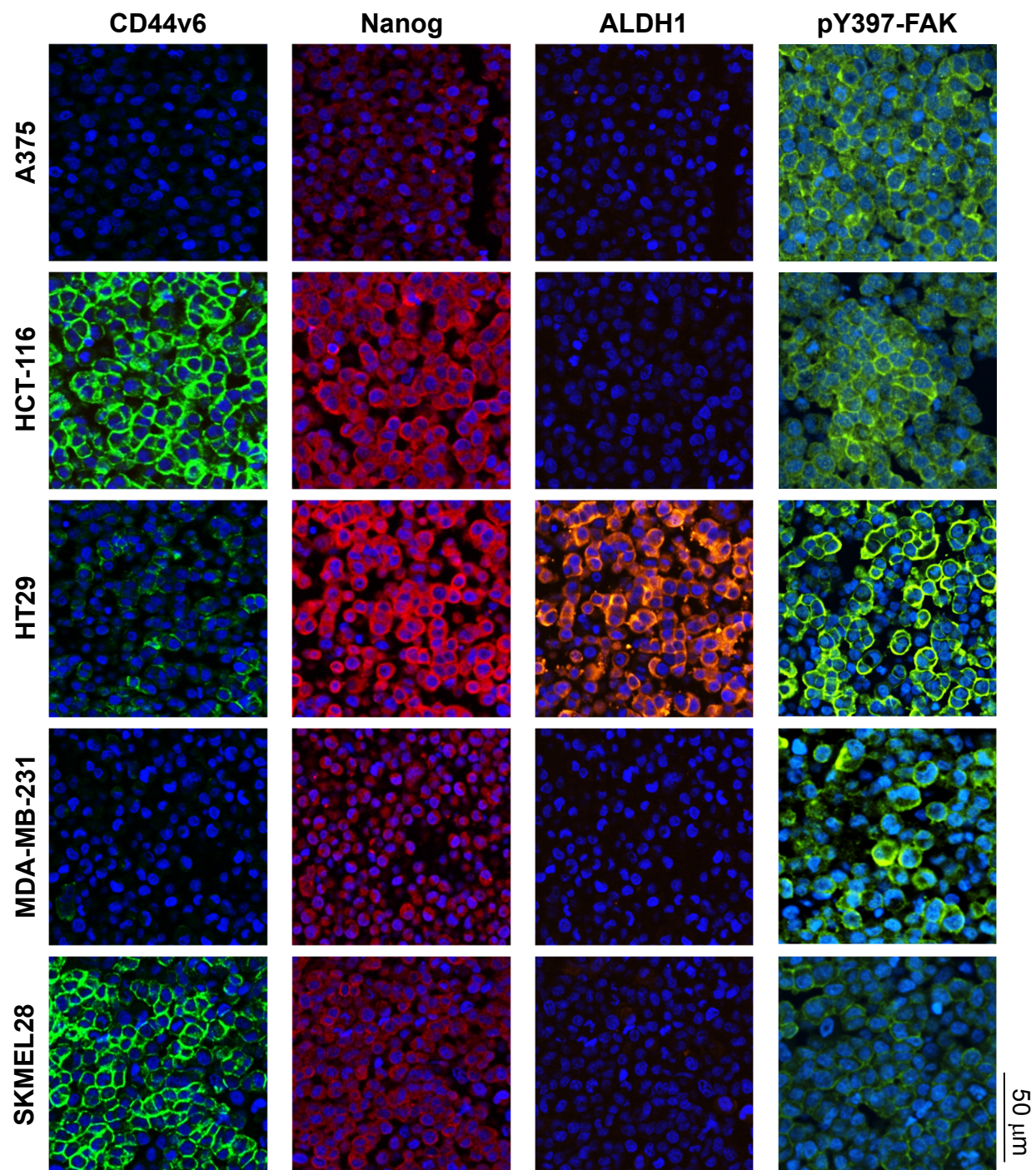

Supplement: S2 Fig — Cell lines (left) were incubated with primary antibodies specific to the indicated CSC-associated proteins (top), followed by the respective fluorescence-conjugated secondary antibodies, to characterize the CSC marker expression in each line (green, CD44v6; red, Nanog; gold, ALDH1; green, pY397-FAK; blue, DAPI; representative 20X images are shown). HT29, which was selected for testing CD133 antibodies due to its high CD133 expression, also exhibits high expression of other CSC markers (Nanog, ALDH1, and pY397-FAK). Primary antibodies used for this experiment include mouse monoclonal anti-CD44v6 (clone 2F10, R&D Systems, Minneapolis, MN), rabbit monoclonal anti-ALDH1 (clone EP1933Y, Abcam, Burlingame, CA), goat polyclonal anti-Nanog (catalog number AF1997, R&D Systems), and rabbit monoclonal anti-phospho-Tyr397-FAK (clone D20B1, Cell Signaling Technology, Danvers, MA). (PDF) [file pone.0199361.s002.pdf]

**Figure S3**

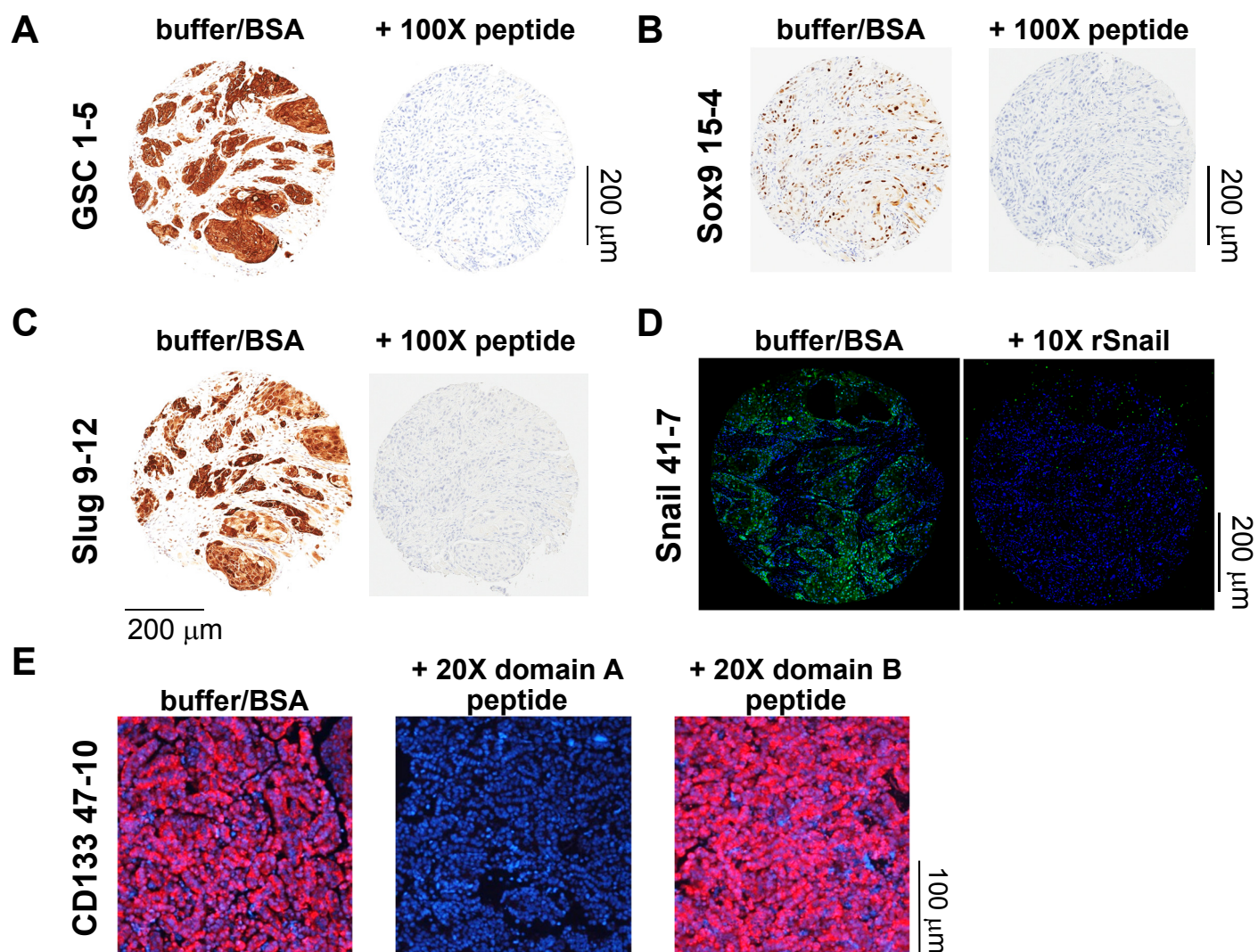

Supplement: S3 Fig — Tumor tissue from homozygous hHGF knock-in mice implanted with H596 NSCLC tumors (A-D) or HT29 cell pellets (E) were combined with antibodies to GSC (A), Sox9 (B), Slug (C), Snail (D), or CD133 domain A (E). In addition, the tissues/cell pellets and antibodies were incubated with either buffer/BSA (left) or peptides/protein corresponding to the target epitope of each antibody (right) at the indicated concentrations relative to that of the antibody (see S3 Table for blocking peptide/protein sequences). Target protein was visualized by immunohistochemistry using HRP-conjugated anti-rabbit antibody (A-C) or by immunofluorescence microscopy using a fluorescence-conjugated anti-rabbit secondary antibody (D-E); representative 20X images are shown. (PDF) [file pone.0199361.s003.pdf]

Figure S4

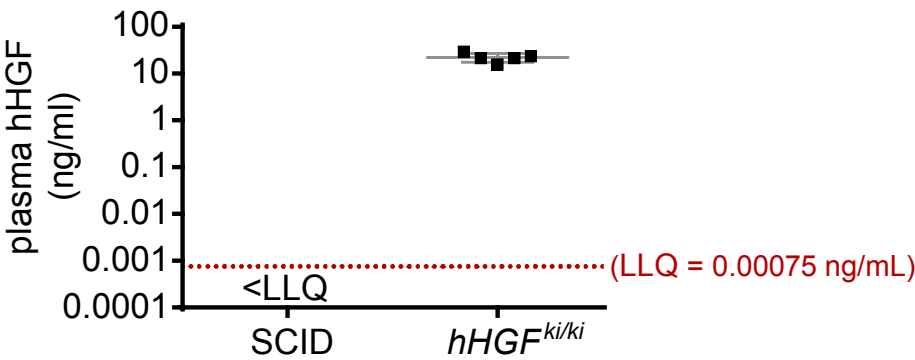

Supplement: S4 Fig — SCID or homozygous hHGF knock-in (hHGFki/ki) mice were implanted with H596 NSCLC tumors (n = 5 animals per group), and tumors were harvested 33 days after implantation. (A) Homozygous hHGF knock-in enhances plasma hHGF levels in H596 tumor−bearing SCID mice. SCID animals exhibited plasma hHGF levels below the lower limit of quantitation (LLQ) of 0.00075 ng/mL (red dashed line); mean plasma hHGF ± standard deviation for hHGFki/ki animals is shown (n = 5). (PDF) [file pone.0199361.s004.pdf]
