# Supplementary material for: Novel antibody reagents for characterization of drug- and tumor microenvironment-induced changes in epithelial-mesenchymal transition and cancer stem cells
Source: PLoS One. 2018 Jun 21;13(6):e0199361. doi: 10.1371/journal.pone.0199361 (PMC6013203; doi:10.1371/journal.pone.0199361)
Supplement: S1 Table — (DOCX) [file pone.0199361.s005.docx]

**S1 Table. EMT and CSC-associated target protein functions and associated malignancies.**

| **Target protein** | **Target protein function^*^** | **Malignancies in which protein is implicated or being investigated^*^** |
| --- | --- | --- |
| GSC | spatial programming (regulates chordin) | malignant glioma |
| Sox9 | skeletal development | chondrosarcoma, synovial sarcoma |
| Slug | represses E-cadherin | breast, pancreatic, squamous cell and various other carcinomas, malignant mesothelioma, osteosarcoma, chronic myeloid leukemia |
| Snail | represses E-cadherin | gastric, breast |
| CD133 | proposed role in apical plasma membrane organization of epithelial cells | leukemia, prostate, retinoblastoma, being investigated in neuroblastoma |

^*^Information obtained from http://www.genecards.org
