# Supplementary material for: Novel antibody reagents for characterization of drug- and tumor microenvironment-induced changes in epithelial-mesenchymal transition and cancer stem cells
Source: PLoS One. 2018 Jun 21;13(6):e0199361. doi: 10.1371/journal.pone.0199361 (PMC6013203; doi:10.1371/journal.pone.0199361)
Supplement: S2 Table — The novel monoclonal antibodies presented in this manuscript are shown in bold for comparison. aa: amino acid; m: monoclonal; n: no; n.a.: no information available on company website; p: polyclonal; y: yes *precise epitope sequence unknown or undisclosed by the company **This column indicates whether antibody has been validated for immunofluorescence microscopy, per company product sheet. †epitope may contain a glycosylation site ‡not validated for use on formalin-fixed or paraffin-embedded tissue, per company product sheet. (DOCX) [file pone.0199361.s006.docx]

**S2 Table. Epitope information for commonly used, commercially available antibodies to EMT TF and CSC proteins (*table continues on next page*).**

| **Target protein** | **Antibody** | **Clonality** | **Epitope/**  **Immunogen** | **Validated for IF**** |
| --- | --- | --- | --- | --- |
| **GSC** | **GSC 1-5** | **m** | **aa 2-18** | **y** |
|  | Abcam EPR4847 | m | undisclosed* | n^‡^ |
|  | Abcam ab86968 | p | aa 36-85 | n |
|  | Invitrogen 1C2 | m | aa 78-186* | n |
|  | Invitrogen OTI1D7 | m | aa 107-257* | n |
|  | Invitrogen OTI4F11 | m | aa 107-257* | y |
|  | Invitrogen PA5-28380 | p | aa 28-257* | n |
|  | Invitrogen PA5-47575 | p | aa 48-159* | y |
|  | Invitrogen PA5-40495 | p | N-terminus* | n |
|  | Millipore Sigma ABD15 | p | C-terminus* | n |
| **Sox9** | **Sox9 15-4** | **m** | **aa 48-66** | **y** |
|  | Millipore Sigma AB5535 | p | C-terminus* | y |
|  | Cell Signaling Technology D8G8H | m | N-terminus* | y^‡^ |
|  | Abcam ab26414 | p | aa 1-100* | n |
|  | Abcam EPR14335-78 | m | aa 150-300* | y |
|  | Abcam EPR12755 | m | aa 400-509* | y |
|  | Abcam 3C10 | m | aa 400-509* | y |
|  | Invitrogen 7H13L8 | m | aa 4-17 or aa-47-62* | y |
|  | Invitrogen 1B11 | m | undisclosed* | y |
|  | Invitrogen PA5-32597 | p | aa 1-150* | n |
|  | Invitrogen PA1-29537 | p | aa 173-185 | n |
|  | Invitrogen PA5-23383 | p | aa 241-257 | n |
|  | Invitrogen PA5-32598 | p | aa 316-510* | n |
| **Slug** | **Slug 9-12** | **m** | **aa 101-116** | **y** |
|  | Cell Signaling Technology C19G7 | m | undisclosed* | y^‡^ |
|  | Millipore Sigma ABE993 | p | unknown* | n |
|  | Abcam mAbcam51772 | m | aa 100-200* | y |
|  | Abcam ab27568 | p | aa 100-268* | y |
|  | Abcam ab183760 | p | aa 120-268* | n |
|  | Abcam ab106077 | p | aa 1-100* | n |
|  | Invitrogen OTI1G7 | m | undisclosed* | y |
|  | Invitrogen OTI1A6 | m | undisclosed* | n |
|  | Invitrogen PA1-86737 | p | center portion* | y |

*(Table continues on next page.)*

**S2 Table (*continued*). Epitope information for commonly used, commercially available antibodies to EMT TF and CSC proteins.**

| **Target protein** | **Antibody** | **Clonality** | **Epitope/**  **Immunogen** | **Validated for IF**** |
| --- | --- | --- | --- | --- |
| **Snail** | **Snail 41-7** | **m** | **aa 22-39** | **y** |
|  | Cell Signaling Technology C15D3 | m | undisclosed* | n |
|  | Millipore Sigma MABE167 | m | N-terminus* | n |
|  | Abcam ab167609 | p | undisclosed* | y |
|  | Abcam ab53519 | p | aa 8-19 | n |
|  | Abcam CL3700 | m | aa 1-28 | n |
|  | Abcam ab82846 | p | undisclosed* | n |
|  | Abcam Sn9H2 | m | undisclosed* | n |
|  | Abcam ab110490 | p | aa 50-150* | n |
|  | Abcam EPR21043 | m | aa 1-264* | n |
|  | Invitrogen 20C8 | m | undisclosed* | y |
|  | Invitrogen F.31.8 | m | undisclosed* | n |
|  | Invitrogen C.775.8 | m | undisclosed* | n |
|  | Invitrogen PA5-23482 | p | aa 3-20 | n |
|  | Invitrogen PA5-11925 | p | aa 9-36 | n |
|  | Invitrogen PA5-23472 | p | aa 87-104 | y |
| **CD133** | **CD133 47-10** | **m** | **aa 295-329** | **y** |
|  | Miltenyi Biotec CD133/1; AC133 | m | “epitope 1”*^†^ | n.a.^‡^ |
|  | Miltenyi Biotec CD133/1; W6B3C1 | m | “epitope 1”*^†^ | n.a. |
|  | Miltenyi Biotec CD133/2; 293C3 | m | “epitope 2”*^†^ | n.a. |
|  | Millipore Sigma MAB4399-I (17A6.1) | m | N-terminus* | y |
|  | Abcam ab19898 | p | aa 800-865* | y |
|  | Abcam EPR20980-104 | m | aa 500-800*^†^ | n |
|  | Abcam EPR16508 | m | aa 250-400*^†^ | n |
|  | Invitrogen 13A4 | m | undisclosed*^†^ | y |
|  | Invitrogen 5E3 | m | undisclosed*^†^ | n |
|  | Invitrogen PA5-38014 | p | C-terminus*^†^ | y |
|  | Cell Signaling Technology D4W4N XP | m | aa 257-281^†^ | n |
|  | Cell Signaling Technology D2V8Q XP | m | aa 303-312 | y |

The novel monoclonal antibodies presented in this manuscript are shown in bold for comparison.

aa: amino acid; m: monoclonal; n: no; n.a.: no information available on company website; p: polyclonal; y: yes

*precise epitope sequence unknown or undisclosed by the company

**This column indicates whether antibody has been validated for immunofluorescence microscopy, per company product sheet.

^†^epitope may contain a glycosylation site

^‡^not validated for use on formalin-fixed or paraffin-embedded tissue, per company product sheet
