# Supplementary material for: Novel antibody reagents for characterization of drug- and tumor microenvironment-induced changes in epithelial-mesenchymal transition and cancer stem cells
Source: PLoS One. 2018 Jun 21;13(6):e0199361. doi: 10.1371/journal.pone.0199361 (PMC6013203; doi:10.1371/journal.pone.0199361)
Supplement: S3 Table — (DOCX) [file pone.0199361.s007.docx]

**S3 Table. Peptides and proteins for blocking experiments with antibodies to EMT- and CSC-associated proteins.**

| **Antibody** | **Amino acid residues for blocking agent** | **Blocking peptide/protein sequence** |
| --- | --- | --- |
| GSC 1-5 | 2-20 | PASMFSIDNILAARPRCKD |
| Sox9 15-4 | 41-67 | SDTENTRPQENTFPKGEPDLKKESEED |
| Slug 9-12 | 101-118 | GSESPISDEEERLQSKLS |
| Snail 41-7 | full-length recombinant Snail | (full-length recombinant protein) |
| CD133 47-10 | 295-329 (“domain A” BSA-conjugated epitope peptide) | KTSLRSSLNDPLCLVHPSSETCNSIRLSLSQLNSN |
|  | 302-313 (“A1” epitope peptide) | LNDPLCLVHPSS |
|  | 317-325 (“A2” epitope peptide) | NSIRLSLSQ |
|  | 615-643 (“domain B” BSA-conjugated non-epitope peptide) | RKNLQDFAACGIDRMNYDSYLAQTGKSPA |
