# Supplementary material for: Novel antibody reagents for characterization of drug- and tumor microenvironment-induced changes in epithelial-mesenchymal transition and cancer stem cells
Source: PLoS One. 2018 Jun 21;13(6):e0199361. doi: 10.1371/journal.pone.0199361 (PMC6013203; doi:10.1371/journal.pone.0199361)
Supplement: S4 Table — Results shown represent the most stringent outcome from experiments performed by 1–3 independent laboratories (aa: amino acid; L: lysate from target protein-overexpressing cells; n: no; n.d.: not determined; R: purified recombinant target protein; Rec.: recombinant; rGSC: full-length recombinant GSC; y: yes). Asterisks indicate antibodies that were selected and validated for preclinical use. 1All antibodies have been deposited under these names in the NCI Clinical Proteomic Technologies for Cancer (CPTC) antibody portal (https://proteomics.cancer.gov/antibody-portal/) and Developmental Studies Hybridoma Bank (DSHB; http://dshb.biology.uiowa.edu/). 2The recombinant protein tested was an isolated domain (see S4 Table). 3A nonspecific band was also observed by Western blot. 4"y" indicates antibodies for which sufficient staining and proper target protein subcellular localization were observed by IFA. 5Protein was detected in nucleus and cytoplasm by IFA. 6For antibodies generated using peptide immunogens, the immunogenic peptide was used for IP-MS. For antibodies generated using rGSC as an immunogen, a peptide containing aa 2-18 was used for IP-MS. (DOCX) [file pone.0199361.s008.docx]

**S4 Table. EMT and CSC antibody clone characterization summary.**

Results shown represent the most stringent outcome from experiments performed by 1-3 independent laboratories (aa: amino acid; L: lysate from target protein-overexpressing cells; n: no; n.d.: not determined; R: purified recombinant target protein; Rec.: recombinant; rGSC: full-length recombinant GSC; y: yes). Asterisks indicate antibodies that were selected and validated for preclinical use.

^1^All antibodies have been deposited under these names in the NCI Clinical Proteomic Technologies for Cancer (CPTC) antibody portal (https://proteomics.cancer.gov/antibody-portal/) and Developmental Studies Hybridoma Bank (DSHB; http://dshb.biology.uiowa.edu/).

^2^The recombinant protein tested was an isolated domain (see Supporting Information Table S4).

^3^A nonspecific band was also observed by Western blot.

^4^"y" indicates antibodies for which sufficient staining and proper target protein subcellular localization were observed by IFA.

^5^Protein was detected in nucleus and cytoplasm by IFA.

^6^For antibodies generated using peptide immunogens, the immunogenic peptide was used for IP-MS. For antibodies generated using rGSC as an immunogen, a peptide containing aa 2‑18 was used for IP-MS.
