# Supplementary material for: Novel antibody reagents for characterization of drug- and tumor microenvironment-induced changes in epithelial-mesenchymal transition and cancer stem cells
Source: PLoS One. 2018 Jun 21;13(6):e0199361. doi: 10.1371/journal.pone.0199361 (PMC6013203; doi:10.1371/journal.pone.0199361)
Supplement: S5 Table — Plasmids encoding these truncated target proteins are available through the DNASU repository https://dnasu.org/DNASU. (DOCX) [file pone.0199361.s009.docx]

**S5 Table. Recombinant protein domain sequences for antibody selection and validation experiments.**

| **Target** | **Clone** | | **Immunogen** | **Recombinant protein domain sequence** |
| --- | --- | --- | --- | --- |
|  |  |  |  |  |
|  |  |  |  |  |
| **FOXC2** |  | 126 | aa 15-33 | aa 1-70 |
| **FOXQ1** |  | 38 | aa 1-22 | aa 1-110 |
|  |  | 46 | aa 1-22 | aa 1-110 |
| **SOX9** |  | 15 | aa 48-66 | aa 1-150 |
|  |  | 35 | aa 437-455 | aa 375-509 |
| **ZEB1** |  | 23 | aa 458-477 | aa 300-510 |
|  |  | 29 | aa 458-477 | aa 300-510 |
|  |  | 44 | aa 697-720 | aa 503-1003 |
| **CD133** |  | 47 | aa 295-329 | aa 180-400 |
|  |  | 133 | aa 615-643 | aa 515-745 |
| **FOXO3** |  | 3 | aa 43-61 | aa 10-170 |
|  |  | 154 | aa 655-673 | aa 355-673 |
| **ZEB2** |  | 69 | aa 809-827 | aa 647-1214 |

## Plasmids encoding these truncated target proteins are available through the DNASU repository https://dnasu.org/DNASU.
